# A Semi-Automatic Method to Extract Canal Pathways in 3D Micro-CT Images of Octocorals

**DOI:** 10.1371/journal.pone.0085557

**Published:** 2014-01-23

**Authors:** Alfredo Morales Pinzón, Maciej Orkisz, Catalina María Rodríguez Useche, Juan Sebastián Torres González, Stanislas Teillaud, Juan Armando Sánchez, Marcela Hernández Hoyos

**Affiliations:** 1 Grupo Imagine – Departamento de Ingeniería de Sistemas y Computación, Universidad de los Andes, Bogotá, Colombia; 2 CREATIS – CNRS UMR5220, Université Lyon 1, Villeurbanne, France; 3 Laboratorio de Biología Molecular Marina (BIOMMAR), Departamento de Ciencias Biológicas – Facultad de Ciencias, Universidad de los Andes, Bogotá, Colombia; University of Zurich, Switzerland

## Abstract

The long-term goal of our study is to understand the internal organization of the octocoral stem canals, as well as their physiological and functional role in the growth of the colonies, and finally to assess the influence of climatic changes on this species. Here we focus on imaging tools, namely acquisition and processing of three-dimensional high-resolution images, with emphasis on automated extraction of canal pathways. Our aim was to evaluate the feasibility of the whole process, to point out and solve – if possible – technical problems related to the specimen conditioning, to determine the best acquisition parameters and to develop necessary image-processing algorithms. The pathways extracted are expected to facilitate the structural analysis of the colonies, namely to help observing the distribution, formation and number of canals along the colony. Five volumetric images of *Muricea muricata* specimens were successfully acquired by X-ray computed tomography with spatial resolution ranging from 4.5 to 25 micrometers. The success mainly depended on specimen immobilization. More than 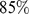 of the canals were successfully detected and tracked by the image-processing method developed. Thus obtained three-dimensional representation of the canal network was generated for the first time without the need of histological or other destructive methods. Several canal patterns were observed. Although most of them were simple, *i.e.* only followed the main branch or “turned” into a secondary branch, many others bifurcated or fused. A majority of bifurcations were observed at branching points. However, some canals appeared and/or ended anywhere along a branch. At the tip of a branch, all canals fused into a unique chamber. Three-dimensional high-resolution tomographic imaging gives a non-destructive insight to the coral ultrastructure and helps understanding the organization of the canal network. Advanced image-processing techniques greatly reduce human observer's effort and provide methods to both visualize and quantify the structures of interest.

## Introduction

Branching patterns are omnipresent in nature, from geological structures such as river drainage networks and glaciers to organic systems such as leaf vessels, and the mammalian blood-vessels and bronchi, among many others [1], [2]. Octocorals of the gorgonian type (*Cnidaria: Octocorallia: Holaxonia*) are marine animals whose polyps grow forming complex tree-like networks. They are basically formed by a holdfast strongly attached to a hard substrate and a main stem, from which branches furcate in a diverse array of patterns [3]. From that simple theme, seafan, pinnate, and candelabrum-like colony forms have evolved multiple times in the octocoral phylogeny, which evoke the labile and plastic nature of branching in octocorals [4]. Indeed, octocoral branching is an adaptive process, where every single colony is different from each other, but seems to follow simple self-organized criticality rules that are species specific and directly related to the species shape and magnitude [5].

There is a remarkable analogy between octocoral networks and the branching organization of plants. Noted for the first time by Leonardo Da Vinci, the number of vessels in a tree trunk is directly related to the number of leaves in the tree crown what is formally known today as the pipe-model theory [6]. Similarly, octocorals have a pseudo-vasculature composed of stem canals, and the number of canals at the main stem is related to the total number of branches in the colony [7].

According to [8], octocoral canals are equally sized vessel-like structures parallel to the gorgonin axis, which extend on the whole colony and have a role related to the water and nutrient exchange between different parts of the colony and polyps. Little is known about actual physiological processes involving these canals, such as their role in calcification and nutrient transport, although the role of the octocoral pseudo-vasculature seems closely related to colony growth and regeneration [9], [10]. In the latter work, octocoral colonies of different sizes were clipped down to an equal-size remaining fragment. Within a fixed period of time the clipped colonies produced different numbers of new branches in proportion to the number of branches present before clipping. This result suggests that new branches from clipped colonies should be directly related to the number of stem canals in the remaining stem.

Despite the expected great importance of stem canals for the colonial arrangement, regeneration and growth of a coral group comprising over three thousand species distributed worldwide, it is also unclear how are their anatomy, organization, skeletal composition and, most important, their configurations at the branching points. All we know at the moment is that it is a complex network spreading throughout the whole colony, that the number of canals decreases towards the branch tips and that the canal wall is densely packed with calcite spindle-like sclerites [7].

Coral reef scientist are facing new environmental challenges, which require the development of new experimental techniques. Constant accumulation of atmospheric 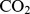 is also altering the seawater chemistry, producing what is known as ocean acidification [11]. Marine organisms with hard skeletons, such as corals, are facing gradually less favorable conditions to deposite calcium carbonate skeletons. Octocorals appear to be more resilient to ocean acidification conditions than scleractinian corals. The few studies on soft corals under these conditions [12], [13] clearly show that octocorals can withstand lower pH values than scleractinian corals. However, it is unknown if other octocorals, such as gorgonian corals, which, as opposed to soft corals, have a gorgonin axial skeleton, can also tolerate ocean acidification. Although the direct effects of ocean acidification on marine organisms are highly variable, there is a generalized negative trend [14]. Of particular concern are coral reef ecosystems, given the concentration of calcifying organisms, which are the main habitat forming species [15]. Accurate predictions on the effects of ocean acidification depend on mesocosm experiments, where seawater chemistry is controlled experimentally [16]. Moreover, methods for measuring short term changes in calcification and skeletal growth are greatly needed. At the moment, there is no method allowing for monitoring skeletal growth without sacrificing the experimental subject.

### Related work

The micro-CT imaging is gaining attention because of its high resolution and non-destructive characteristics. In this context, “non-destructive” refers to the fact that the specimen can be reused after image acquisition, as opposed to such techniques as cutting it into tiny slices, in order to observe its micro-structure. Previously, standard CT images were used to evaluate the form and growth of sponges and corals [17], [18]. More recent studies have used micro-CT images to analyze and characterize the internal and external structure of corals [19]–[23] and sponges [24], [25]. Some of those investigations treated corals as bone-scaffold material for clinical implants, with the aim to asses the compatibility of structural, mechanical, physical and biological properties [19]–[21]. Other studies aimed at understanding the internal and external morphology, its relationship with the physiological functions of the colony (growth, circulation of nutrients...) and the impact of climatic changes on these animals [22], [23].

To assess the usefulness of corals as scaffold material, the average internal diameter of ducts was manually measured in two species: stony coral (*Scleractinia*) and velvet finger (*Montipora digitata*) [20]. As a manual three-dimensional (3D) analysis of micro-CT images is tedious and arduous, various image processing methods have been used in order to facilitate the visualization of the structures of interest and/or to partly automate the analysis. Hereafter, we focus on previous work that has used image-processing techniques to partly automate some measurements. A threshold-based image segmentation was used in [21] to compare the porosity, pore size and mechanical properties of three species: *Acropora*, *Goniopora* and *Porites*. Strucutural properties measured in segmented images and physical properties deduced from numerical simulation were also used to compare *Acropora* and *Porites* [19]. As the partial volume effect occurring at the image-acquisition and image-reconstruction stages hampers the definition of the borders between phases, the image segmentation was preceded by a multi-step filtering based on anistropic diffusion.

The skeletal density and porosity of *Acropora pulchra* was measured to assess the effects of anthropogenic influences on corals [22]. The measurements were obtained from images processed in two steps: 1) correction of the beam hardening effect, and 2) segmentation by an adaptive thresholding method. Based on a visual inspection, the internal network architecture of an Indonesian *Stylaster* was described and its growth model was proposed [23]. Thresholding was used, combined with mathematical morphology techniques (watersheds, distance transform, skeletonization) provided by a software tool called Pore3D [26], in order to quantify the porosity and the average diameters of the canals within two small subvolumes. Another study has used a seeded-region-growing segmentation technique and focused on the skeletonization of coral branches, so as to evaluate the tree-like structure of a whole *Madracis mirabilis* colony [18]. However, this approach is rather macroscopic, as it extracts one axis (skeleton) per branch, while ignoring the internal structure.

The morphological skeletonization used to quantify the canals [23] is also unsuited for our study. Unlike the existing work, we tackle the extraction of the canal pathways in their whole extent, while avoiding spurious skeleton branches due to the local lack of contrast between the canals and the neighboring structures. Our problem is more similar to the extraction of blood vessels in 3D medical images. Many methods have been published, which deal with 3D vascular image segmentation. According to recent surveys [27]–[30], a centerline extraction is an essential step towards the segmentation of such tubular structures as blood vessels. Most existing methods have used an underlying model that enforces the continuity and the smoothness of the centerline, as well as various “tubularness” criteria that are expected to enhance the points close to the centerline of the vessel. These criteria exploit such measures as the gradient flux, the eigenvalues of the inertia matrix or of the Hessian matrix. Usually, such criteria assume that the image intensity (tissue density) is homogeneous along the centerline and strongly varies in the directions perpendicular to the centerline. However, as it will be explained later, these criteria are not directly applicable to octocoral canals.

### Objectives

The long-term goal of our study is to understand the internal organization of the octocoral stem canals, as well as their physiological and functional role in the growth of the colonies. In this article, we focus on imaging tools giving access to the internal ultrastructure and organization of the network. Namely we address 3D micro-CT image acquisition and image processing, with a particular emphasis on automated extraction of canal pathways. By “pathway” we understand a line located within the canal. We use the word “pathway” and avoid the word “centerline”, as the centering is not essential in our application. Our aim was to evaluate the feasibility of the whole process, to point out (and solve if possible) the technical problems related to the conditioning of specimens, to determine the best acquisition parameters and to provide a support for the development of necessary image-processing algorithms. The pathways extracted are expected to facilitate the structural analysis of the colonies, namely to help observing the distribution, formation, and number of canals along the colony, and finally to assess the influence of climatic changes on this species.

## Materials and Methods

### Specimens and images

It is technically unfeasible to acquire one single image representing a whole colony with a sufficient spatial resolution. Furthermore, even if it was feasible, the resulting image size would not be manageable on a standard personal computer. Therefore, the best trade-off was searched between the image resolution and field-of-view (FOV). We conjecture that, if the whole extent of the colony is covered by a collection of smaller overlapping images, appropriate image-processing techniques can align the partial images and subsequently retrieve useful global characteristics of the colony, provided that the spatial location of each image is known with sufficient precision and that the specimen is appropriately immobilized during the acquisition.

#### Specimen preparation

Four specimens of *Muricea muricata* were taken in Colombian territorial waters of the Caribbean Sea (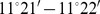 N and 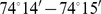 W) in accordance with the collection permits issued by Natural National Parks of Colombia (permit number: DTC-CR-T-036-03/09). The specimens were initially placed in plastic bags filled with marine water. Then they were rinsed with fresh water, fixed with a 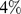 formaline solution, stored in a container filled with alcohol at 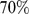 and transported to Lyon, France, where the image acquisitions were performed at the MATEIS laboratory, National Institute for Applied Sciences (INSA). Several samples were prepared for the purpose of image acquisition, in order to empirically determine the optimal configuration.

**Figure 1:**
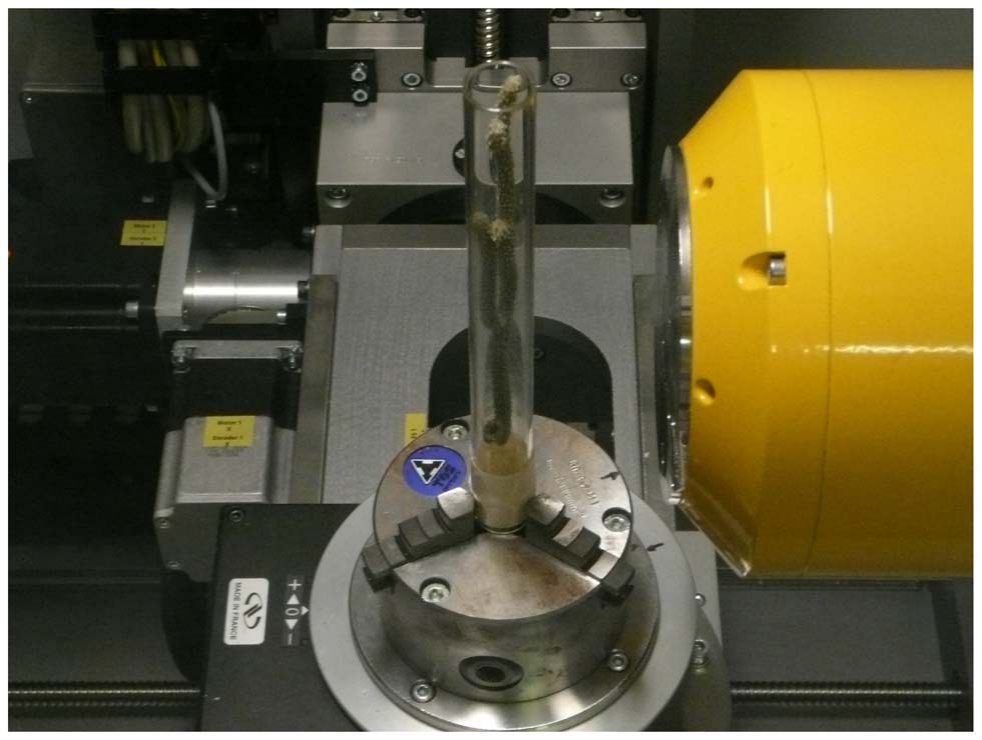
Specimen packed in a tube and placed within the micro-CT imaging device.

The micro-CT image acquisition takes approximately 45 minutes per sample, the temperature within the imaging device may increase up to 40°Celsius and the sample rotates in the center of the device. Therefore, we had to tackle such problems related to the behavior of the samples as: drying, undesirable micro-movements and appearing/disappearing of branches that enter and come out of the FOV during the rotation. To cope with these problems, the samples were conditioned in closed plastic tubes (Fig. 1), some branches were folded along the tube, others unfortunately had to be cut.

#### Image acquisition

**Figure 2:**
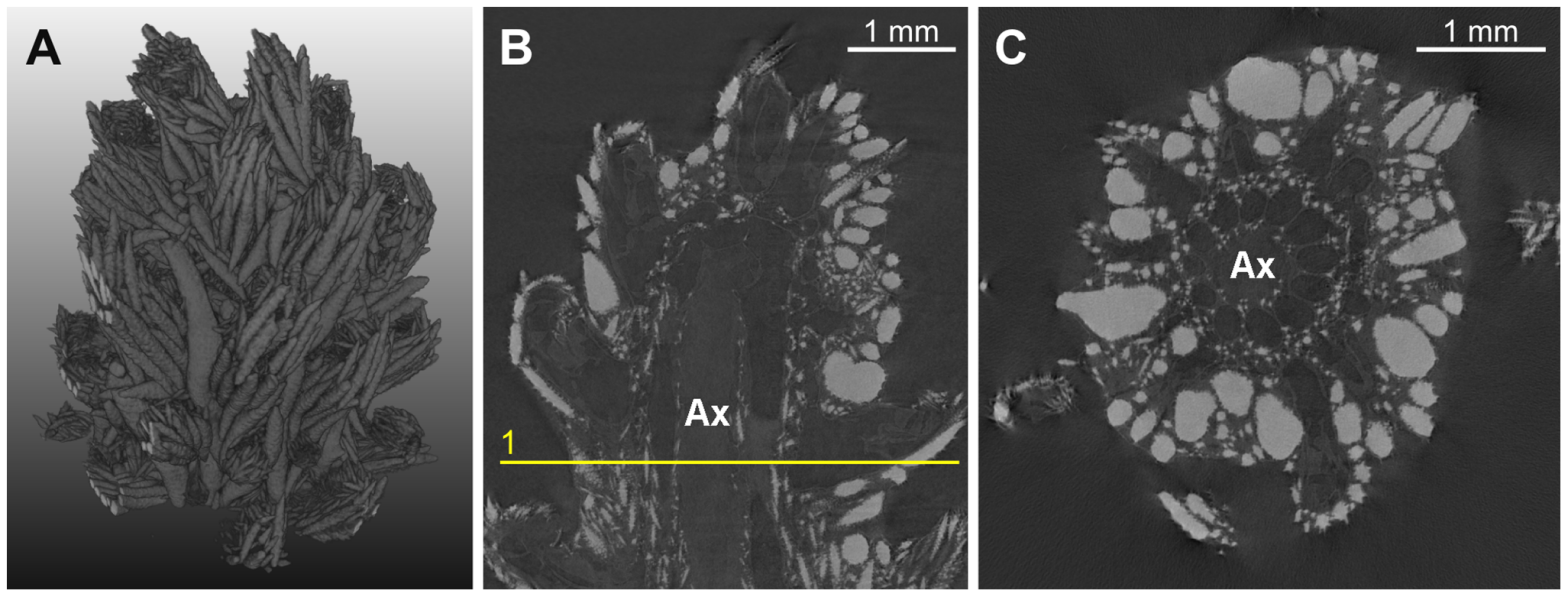
EX dataset. (A) volume rendering, (B) example of longitudinal slice, (C) axial slice corresponding to the initialization plane. The line superimposed onto the image represents the location of the initialization plane. The gorgonin axial skeleton was marked “Ax” and the calcite sclerites appear as bright spots of variable size and shape.

**Figure 3:**
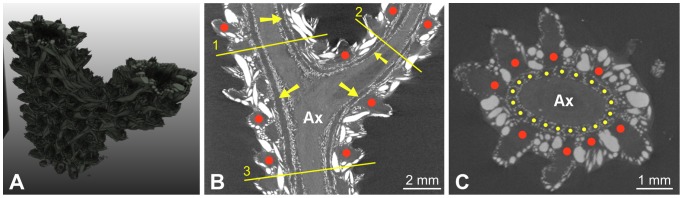
M5 dataset. (A) volume rendering, (B) example of longitudinal slice, (C) axial slice corresponding to the lowest initialization plane. The lines superimposed onto the image represent the location of the initialization planes. The gorgonin axial skeleton was marked “Ax” and the calcite sclerites appear as bright spots of variable size and shape. Examples of polyps were highlighted by red dots. Canals were indicated by yellow arrows in the longitudinal view and by yellow dots in the cross-sectional view.

**Figure 4:**
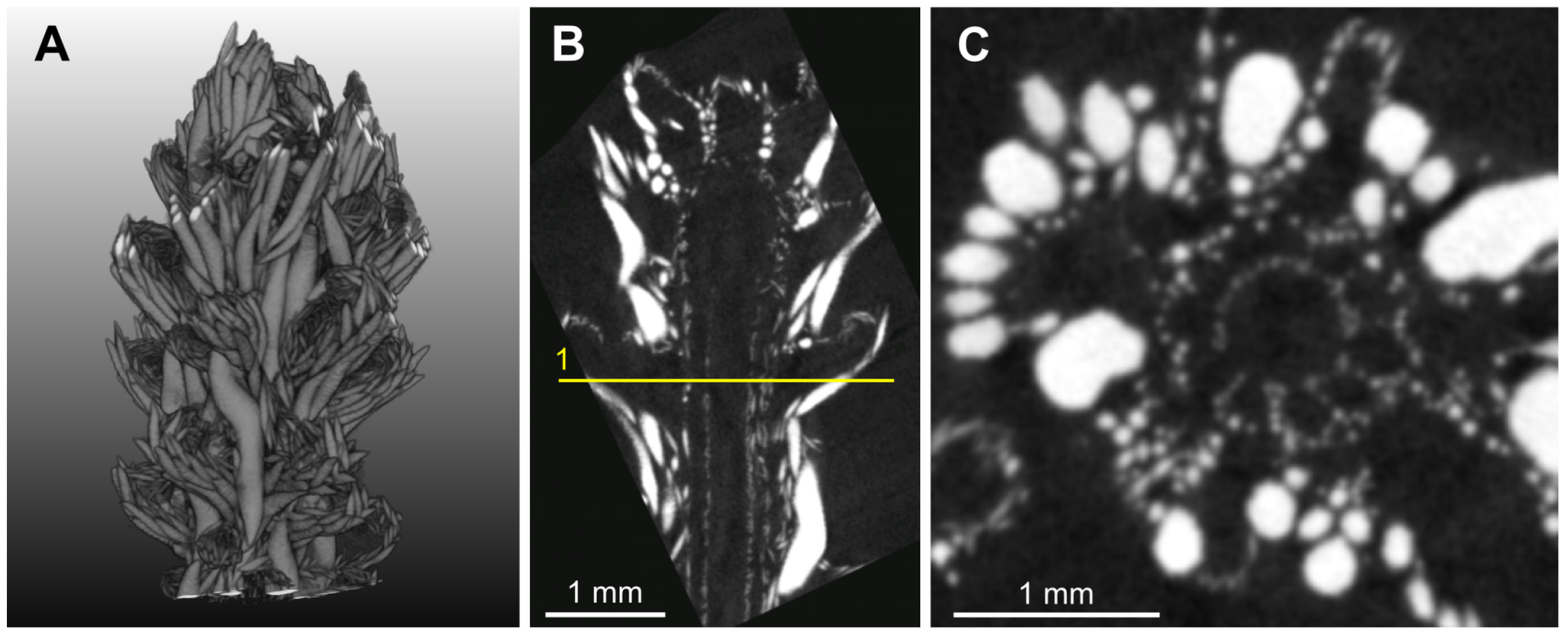
M1 dataset. (A) volume rendering, (B) example of longitudinal slice, (C) axial slice corresponding to the initialization plane. The line superimposed onto the image represents the location of the initialization plane.

**Figure 5:**
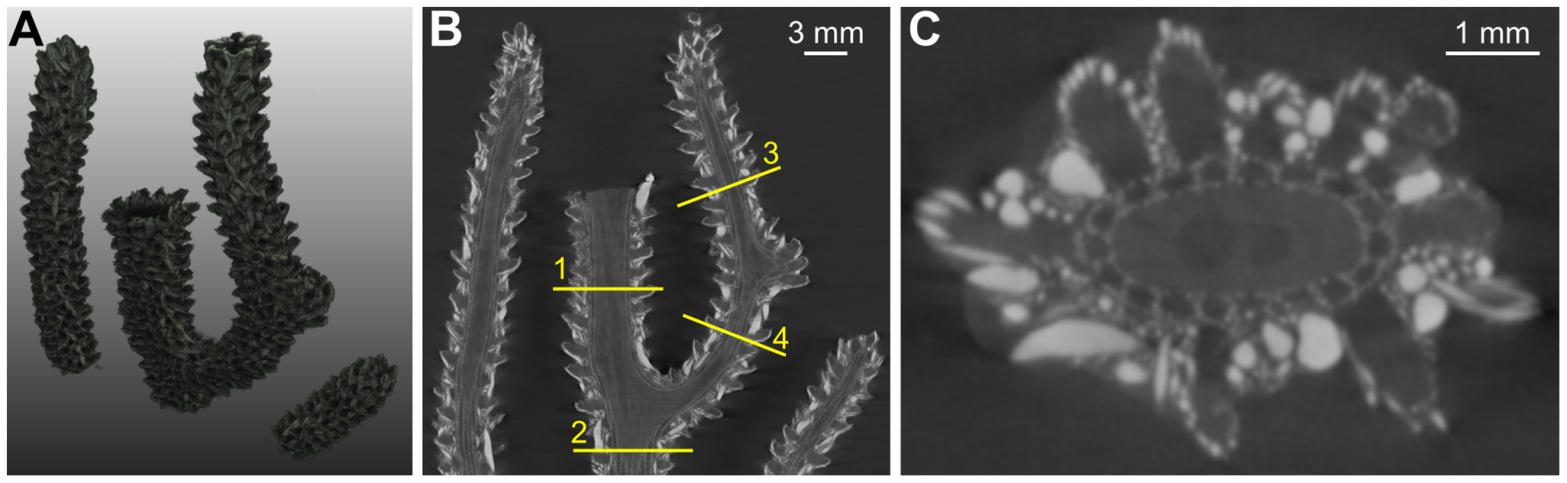
S3 dataset. (A) volume rendering, (B) example of longitudinal slice, (C) axial slice corresponding to the lowest initialization plane. The lines superimposed onto the image represent the location of the initialization planes.

**Figure 6:**
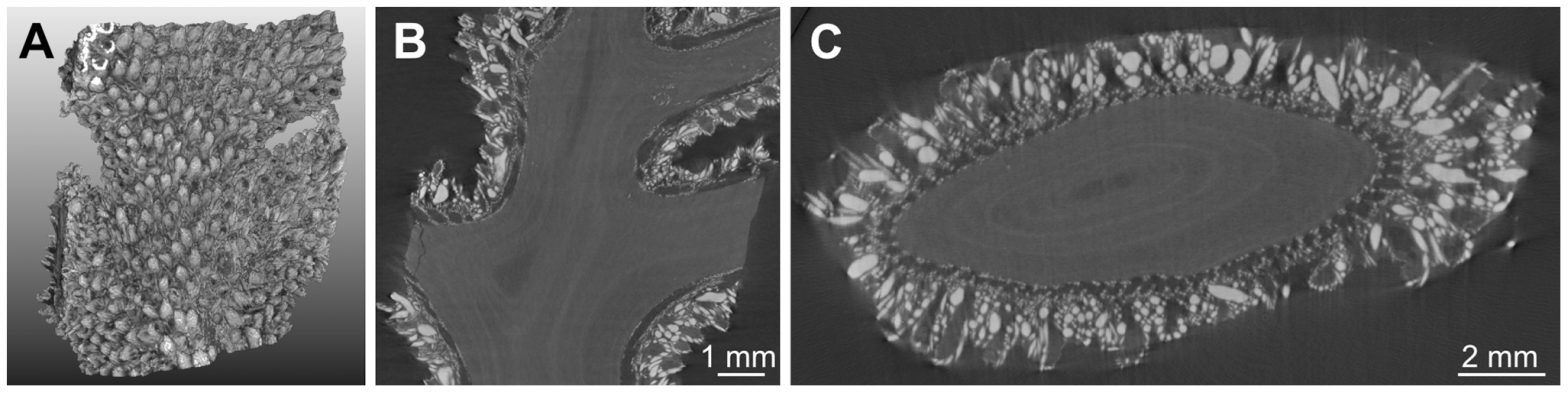
BASE dataset. (A) volume rendering, (B) example of longitudinal slice, (C) example of axial slice.

The images were acquired using a Phoenix X-ray micro-CT device, type vtomex, equipped with a nanofocus tube and a Varian PaxScan 2520 detector. The settings were as follows: voltage from 60 to 80 kV, current 200 to 250 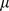
A, exposure time 500 ms, number of projections from 650 to 900. Various FOV/resolution combinations were tested with different samples. The images were reconstructed with isotropic spatial resolutions. Owing to a long duration of each acquisition and to a restricted availability of the imaging device, nine acquisitions were achieved. Five images were judged exploitable. Their parameters are listed in Table 1, while their appearance is illustrated by Figures 2, 3, 4, 5 and 6. The remaining images were contaminated by various artifacts mainly due to an imperfect immobility of the samples. While the total extent of the specimens exceeded 20 cm, the FOV of the images acquired only included small portions ranging from 5 to 32 mm. The image referred to as BASE (Fig. 6), as well as the long straight isolated branch in S3 dataset (on the left in Fig. 5) were used to train the image-processing algorithms, whereas the remaining branches in S3, as well as the images EX, M1 and M5, were used to evaluate these algorithms.

**Table 1:** Parameters of the image acquisitions.

Name	Location	Resol. (*μ*m)	Size (# pixels)			Project.	Voltage (V)	Current (*μ*A)
			X	Y	Z	(#)		
EX	apex	4.5	1000	1000	1200	650	60	200
M5	bifurcation	10	1250	700	1000	900	80	200
M1	apex	15	300	300	450	900	80	250
S3	several	25	1300	300	1300	900	60	200
BASE	trunk	14	1400	700	1300	900	60	200

The design of image processing algorithms was based on several characteristics determined by visual inspection of the images. The reader can particularly refer to Figure 3, where examples of anatomical structures are annotated. Firstly, the gorgonin axis appears as a relatively dark and homogeneous (albeit noisy) region in the center of each branch. It is surrounded by the region of canals that forms a ring in cross-sectional views. Secondly, the canals are more or less parallel as long as a single ramification, far from a branching point, is considered. The canal cross-sections have approximately circular or elliptical shapes with similar diameters. Thirdly, while the interior of the canals is dark and homogeneous, their boundaries are globally clearer but very irregular, as they include numerous small sclerites. Except the finest-resolution image of the apical region, no density differences other than those due to the sclerites were noticed on the boundaries between the canals. This means that the image evidence is not sufficient to distinguish apparent holes (spaces between sclerites) from actual passages (if any) from one canal to another. Lastly, the relatively periodic pattern of the canal region is sometimes disturbed by a large and very bright (dense) sclerite.

### Image processing

The method can be subdivided into three distinct steps: initialization, pathway tracking and post-processing. The core of the approach proposed is an algorithm designed to extract the pathway of a canal, from a starting point located in this canal, until a stopping criterion is reached. As dozens of canals can be present in a single image volume, the initialization step was devised to automate the localization of the starting points in each canal. The architecture of the canal network proved rather complex, so that the initialization needs to be performed at least in each ramification of the colony. However, with such a strategy, some pathways may be extracted twice or more. Furthermore, for various reasons, the stopping criteria may fail. The role of the post-processing step is therefore two-fold: to detect and cut the line segments, the behavior of which is erratic compared to other nearby pathways, on the one hand, and to detect and fuse the lines that follow the same canal, on the other hand. All the image-processing algorithms and the graphical user interface have been implemented using the CreaTools software development platform (http://www.creatis.insa-lyon.fr/site/en/CreaTools).

#### Pathway extraction in a canal

We adopted a tracking strategy inspired by an algorithm that extracts blood-vessel centerlines in magnetic resonance angiography (MRA) images [31]. In that work, the tracking was performed independently in two opposite directions from a starting point. The starting point was interactively selected and next points were added in a prediction/correction scheme. The prediction used the local vessel orientation inferred from the eigenvectors of the inertia matrix. The correction (repositioning) of the new point used the centroid information and was restricted by continuity (elasticity) and smoothness (flexibility) constraints.

**Figure 7:**
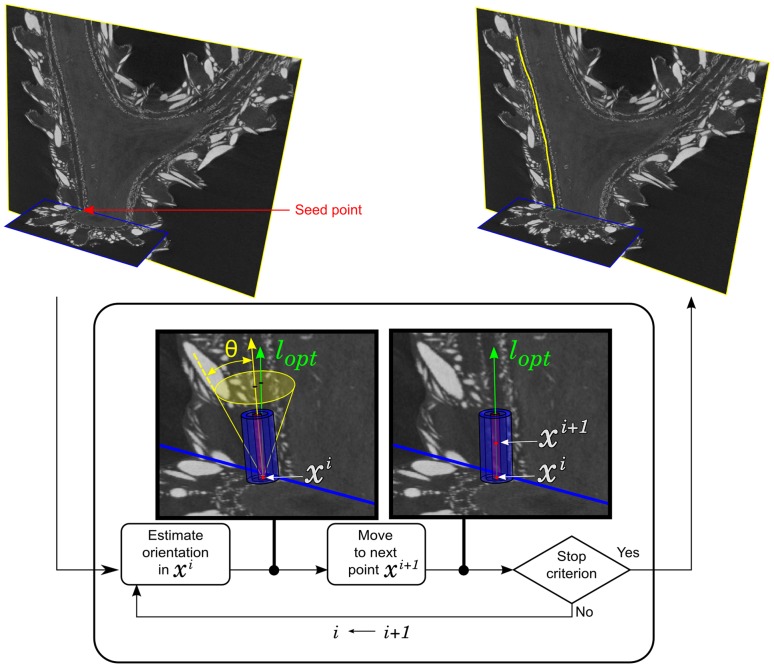
Flowchart of the pathway extraction algorithm. The yellow arrow indicates the previous orientation, while the green one represents the currently found optimal orientation.

Our algorithm (Fig. 7) also iteratively builds the canal pathway, starting from a seed point, until a stopping criterion is met, and each new point is added according to the locally estimated orientation. However, compared to the seminal work, our algorithm mainly differs in the criterion used to estimate the local orientation. During the development, we also found that the repositioning step became unnecessary, as the method adopted to estimate the local orientation proved robust and the predicted points fell into the canal “lumen”, whereas the failures were due to other factors.

The orientation estimates based on the inertia matrix proved unreliable, owing to the highly irregular shape and densities at the boundaries of the canals. For the same reason we rejected the alternative orientation estimates and “tubularness” criteria from the literature, based on derivatives, namely on gradient flow or on the eigen-analysis of the Hessian matrix, which are even more sensitive to such irregularities. Instead, we drew our inspiration from the HD filter that was originally devised to improve the visualization of small vessels in MRA [32]. The first stage of the original HD filter estimated the local orientation of the vessel, while the second stage performed a directional smoothing or contrast enhancement [33]. Here, we are interested in the first stage that seeks, within a set of 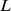 different discrete orientations, the one that optimizes a criterion combining the longitudinal homogeneity (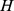) and the radial difference (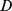) of the image gray levels.

**Figure 8:**
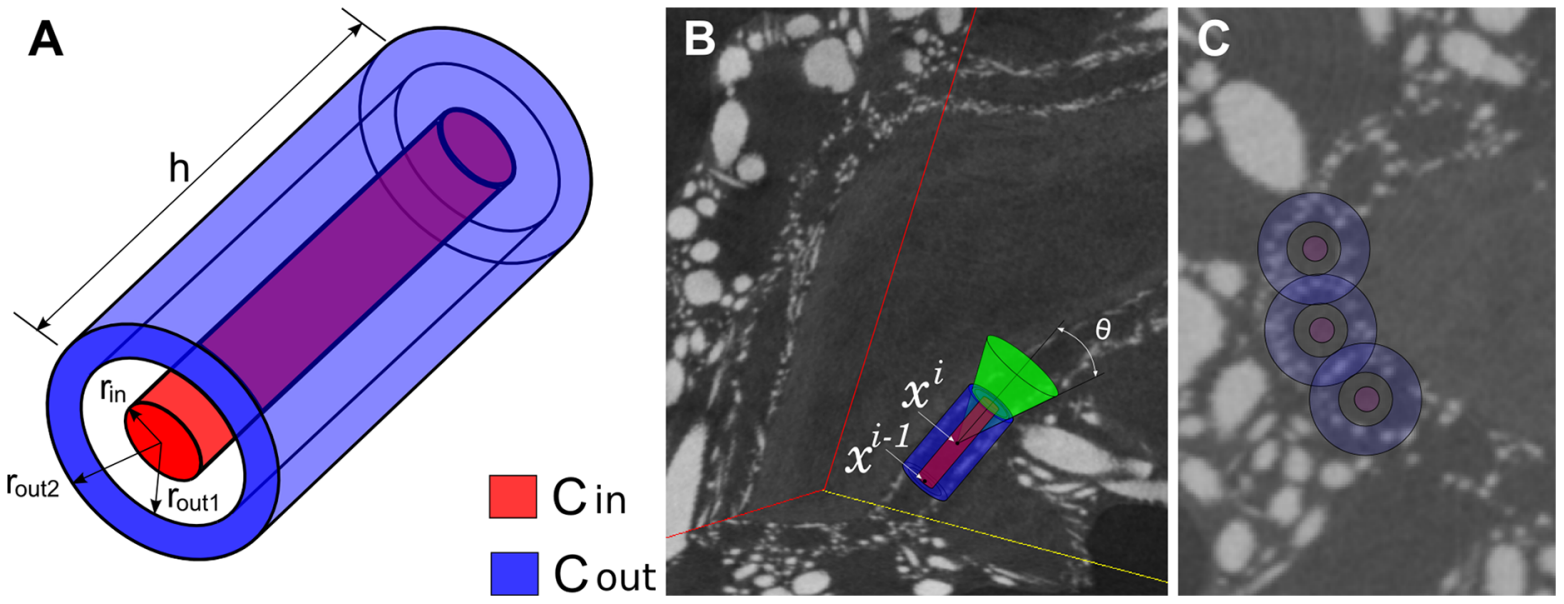
Cylinders used to estimate the local orientation. (A) 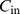, 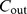 and their dimensions. (B) 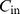, 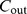 aligned with a canal at the previous point 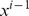, and the cone used to seek for the current orientation. Note the definition of the angle 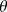 and the location of the current point 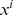 at 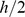 from 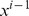. (C) Disks and rings corresponding to 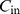, 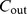 of three neighboring canals in the initial cross-sectional plane.



In the seminal filter, 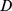 was defined as the mean gray-level difference between two coaxial straight structures: a thin segment expected to coincide with the vessel lumen, and a hollow discrete “pipe” surrounding it. 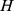 was deduced from the average of standard deviations in both structures. However, while in MRA images it is legitimate to expect that the intensity variations along the vessel boundary be small, the variability along the canals is very large. Therefore, although we adopted the idea of two coaxial structures, we restricted the calculation of 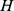 to the internal one. Actually, we used two coaxial cylinders of the same height 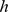: the internal one denoted 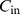 was solid with radius 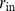 and the external one, denoted 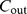, was hollow with inner and outer radii respectively denoted 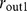 and 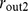 (Fig. 8(a)). The choice of the actual values of 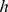, 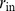, 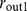 and 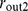 will be specified later. Let 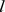 denote the 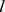-th discrete orientation, and 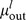, 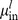 and 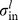 respectively denote the mean values within 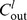 and 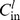, as well as the standard deviation within 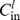, using image gray levels (actually tomographic densities) normalized between 0 and 1. We then have 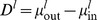 and the criterion 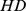 is equal to:
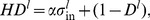
(1)where 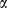 is a weighing coefficient, the value of which was empirically set to 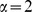. All the empirical parameter settings were obtained during the algorithm development, by successive trials and corrections, using the training data. The local orientation 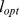 of a canal is estimated by minimizing 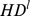:

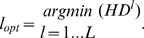
(2)The discretization of the search space is done as follows. Given the current point 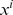 of the pathway and the current orientation (estimated in the previous iteration) we fix in this point one end of the common axis of 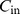 and 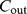. Then the axis sweeps a right cone (Fig. 8(b)) with angular steps of 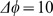 degrees (azimuth) and 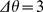 degrees (zenithal distance) around the current orientation. The opening angle 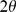 of the cone determines the flexibility (smoothness) of the pathway. This parameter was empirically fixed to 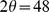 degrees, so that the pathway can locally deviate from a straight line by 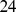 degrees at most. We thus evaluate 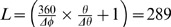 discrete orientations.

Once the local orientation 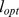 estimated, the location of the next point is predicted along this orientation. There is no particular reason to vary the distance between consecutive pathway points, as neither the diameter nor the curvature of the canals change abruptly. We therefore conservatively set a constant step equal to half the height of the cylinders. This means that the predicted next point 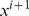 of the pathway is located at a distance equal to 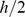 from the current point 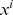 (Fig. 8(b)), according to the local orientation 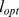: 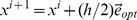, where 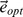 is a unit vector representing the orientation 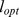.

The tracking stops when the cylinders reach the image boundary or the difference between 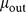 and 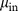 becomes too small. We empirically fixed the latter stopping criterion as follows: 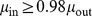.

The initial orientation of the pathway is set orthogonal to the cross-section used to find the starting points. The initialization of the cylinder dimensions is based on 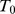, an estimate of the distance between the centers of the neighboring canals. When the seed points are provided manually, 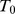 is calculated by averaging the distances between the seeds. In the case of an automated seed determination, based on finding a spatial frequency 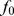 of the gray-level variations, 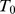 is calculated as the corresponding period 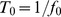 (see File S1, Section 1.3). The inclusion of sclerites in the internal cylinder should be avoided, so the radius of 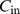 is to be much smaller than 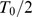. This can be achieved by setting 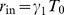, with 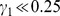. Conversely, 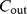 should mainly contain the canal wall, which requires 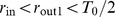. Furthermore, two neighboring canals partly share the same wall, so the corresponding external cylinders may partly overlap (Fig. 8(c)). This can be achieved with 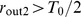. We therefore propose to set these radii as follows: 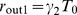, with 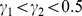, and 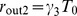, with 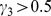. Additionally, the height of the cylinders is set proportional to the radii of 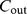: 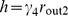. The actual coefficient values 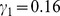, 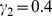, 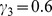 and 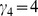 have been determined empirically.

#### Automated determination of the seed points

**Figure 9:**
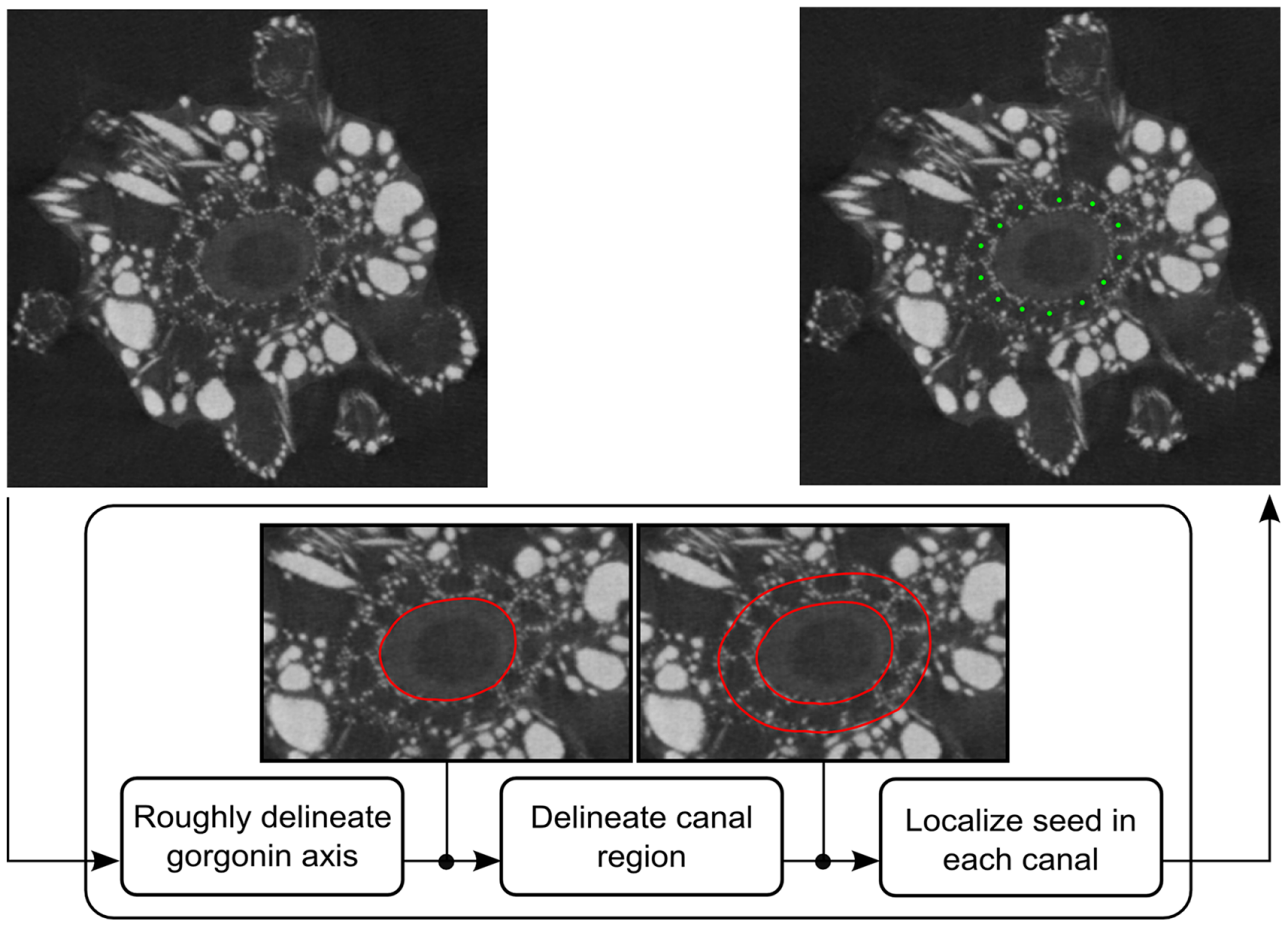
Flowchart of the automated seed-localization algorithm.

As mentioned in the previous section, the pathway tracking requires a starting point within each canal. In medical imaging, an interactive selection of a starting point is often performed. This solution can also be adopted in our application. However, due to the large number of canals to be extracted, some automation is desirable. We developed a pragmatic solution, where all the processing is nearly automatic, but some interactive help can be provided in case of failure. The process begins by selecting one axial cross-section in each branch of the octocoral, located halfway between the ends of the branch (Figs. 2–5). The subsequent steps (Fig. 9) are performed in these cross-sections. The main idea is to delineate a ring around the gorgonin axis, which contains the canals, and to take advantage of the quasi-periodic gray-level variations within this ring, in order to place the starting points in dark “holes” representing the canals. The ring delineation mainly uses mathematical morphology operations, whereas the analysis of the gray-level variations within it uses the Fourier transform to determine the spatial frequency of these variations. The details are provided in File S1, Section 1, and illustrated by Figures S1, S2 and S3.

#### Post-processing

**Figure 10:**
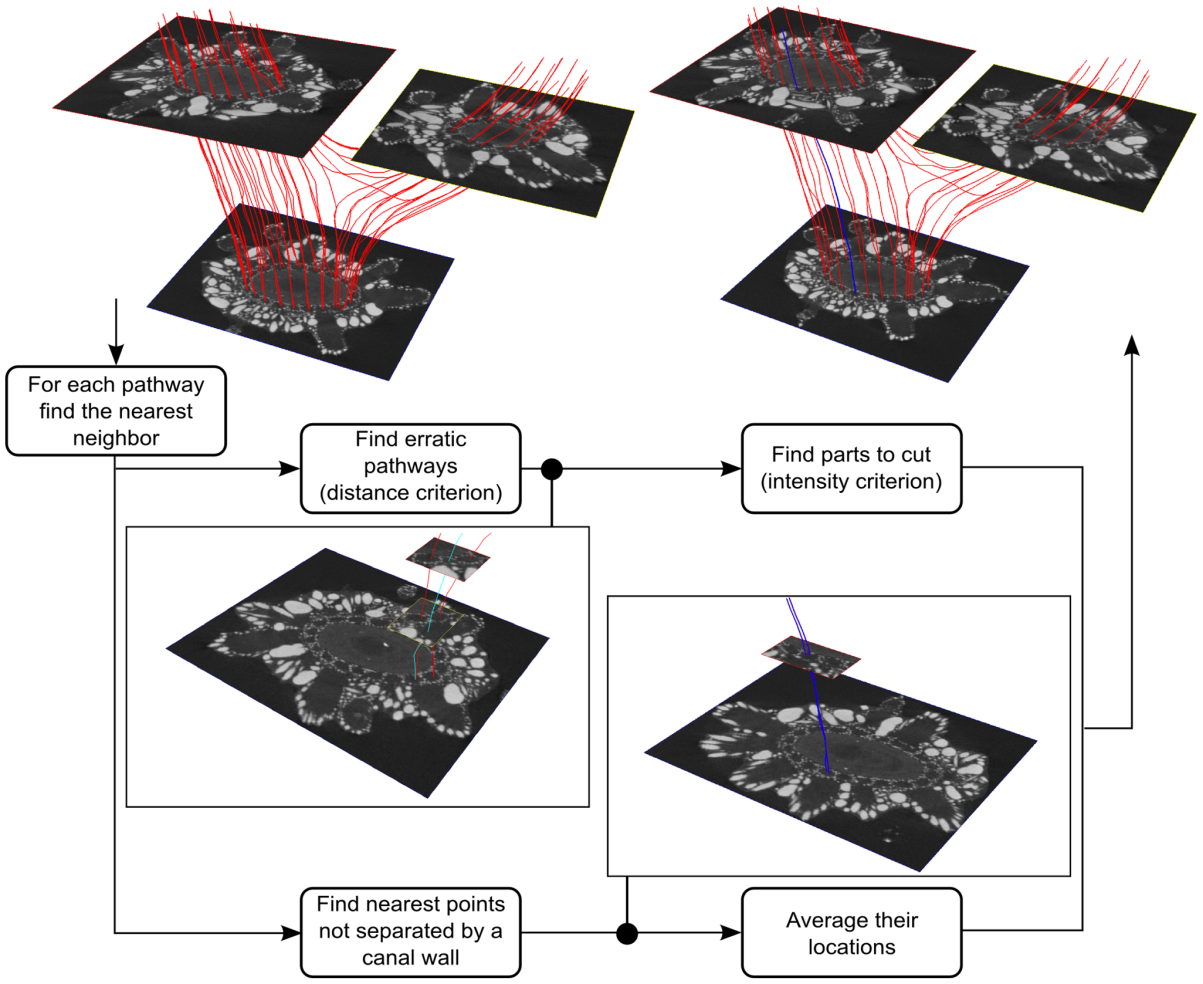
Flowchart of the post-processing.

The goal of the post-processing is to “clean” the set of the pathways extracted: cut their erratic parts, if any, and combine the pathways located within the same canal into a single pathway. It is very unlikely to find an isolated canal without other almost parallel canals nearby. Therefore, when a part of a pathway extracted is too far from the neighboring pathways, this part is probably erratic and should be cut. Our algorithm determines the average distance between each pathway extracted and its nearest neighbor. Then it identifies the pathways, for which this distance is significantly larger than the mean distance observed in the whole set. Within each of thus detected outlying pathways the actually erratic parts are determined by analyzing the gray-levels of the pathway points. Points that significantly deviate from the mean gray-level are removed. Two pathways are located within the same canal if they are not separated by a canal wall. This is detected by analyzing the gray-level profiles between the points of the neighboring pathways. The locations of such point pairs are averaged. These steps (Fig. 10) are detailed in File S1, Section 2, and the profile analysis is illustrated by Figure S4.

### Evaluation Methodology

To evaluate the methods proposed, the pathways automatically extracted were compared to reference pathways. As ground truth was not available, the reference pathways were interactively drawn by a trained observer. In order to assess the accuracy of the pathway extraction algorithm itself, while avoiding possible errors induced by incorrect locations of automatic seed points, the algorithm was initialized with manually selected points. Separately, we evaluated the accuracy of the automatic detection of seed points within canals, in the cases where this option was exploitable. Four datasets (EX, M1, M5 and S3) were used in the evaluation. The dataset BASE, as well as the long straight isolated branch in S3 (on the left in Fig. 5) were left out, since they had previously been used to train the algorithms during their development. In this section, the methodology to assess the pathway-extraction algorithm is first described, and then the evaluation of the automatic detection of starting points is explained.

### Accuracy of the pathway extraction algorithm

A custom-made tool, based on MeVisLab (http://www.mevislab.de/), has been used to manually mark the pathway points along all the perceptible canals. A careful tracing of the pathways was very time-consuming and required several iterations to cope with unexpected canal bifurcations and endings.

Generally speaking, a perfectly extracted pathway should be entirely located within an actual canal and its extent should be equal to the canal length. To evaluate how well the extracted pathways meet these requirements, each of them was compared to the reference pathways. Namely, the following criteria were evaluated:

- how many extracted pathways were correct (label 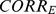), *i.e.* had a corresponding unique reference pathway?
- how many extracted pathways were erratic (label 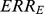) and thus did not correspond to any reference pathway?
- how many reference pathways were missed (label 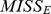), *i.e.* had no corresponding reliable extracted pathway?
- what was the length of the sections, where the extracted and reference pathways overlap, as compared to the total length of these pathways?
- what was the mean 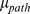 and standard deviation 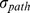 of the point-to-point distances between the extracted and reference pathways in these sections?

The latter two criteria were based on a point-to-point correspondence between the reference and extracted pathways. The details are provided in File S1, Section 3, and illustrated by Figure S5. The proportion of correct pathways among all the extracted ones was defined as *precision*:
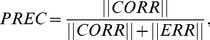
(3)while the ratio of correctly extracted pathways to the total number of reference pathways was defined as *recall*:
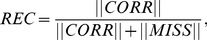
(4)where 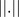 denotes the number of elements in a given label-set. The overlap between the extracted and reference pathways was measured using the *Dice similarity index* (File S1, Eq. 3). This index, as well as the precision and recall, ideally should be close to 1.

### Accuracy of the automatic starting-point detection

A trained observer manually selected reference points (one per canal) near the center of each canal perceptible in each initial cross-section. The automatic seed points were compared with these reference points in two steps.

First, the automatic points were labeled as correct (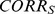) or incorrect (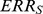), based on a visual inspection. Points were considered as correct if they were located within a canal “lumen” and incorrect otherwise (outside the region of canals or in the sclerite wall separating two adjacent canals). If two automatic points were inside the same canal, the one nearest to the reference point was marked as correct and the other one as incorrect. If there was no automatic seed in a canal, the corresponding reference point was labeled as missed (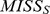). The proportion of correct automatic seed points was calculated according to the definitions of precision and recall (Eqs. 3–4).

Second, the distance between each correct automatic point and the corresponding reference point was calculated. The mean 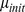 and standard deviation 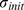 of those distances were computed and compared with the estimated average canal radius 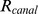, defined as half of the average in-plane distance between the initialization points used in the given plane.

## Results

### Pathway extraction accuracy

**Figure 11:**
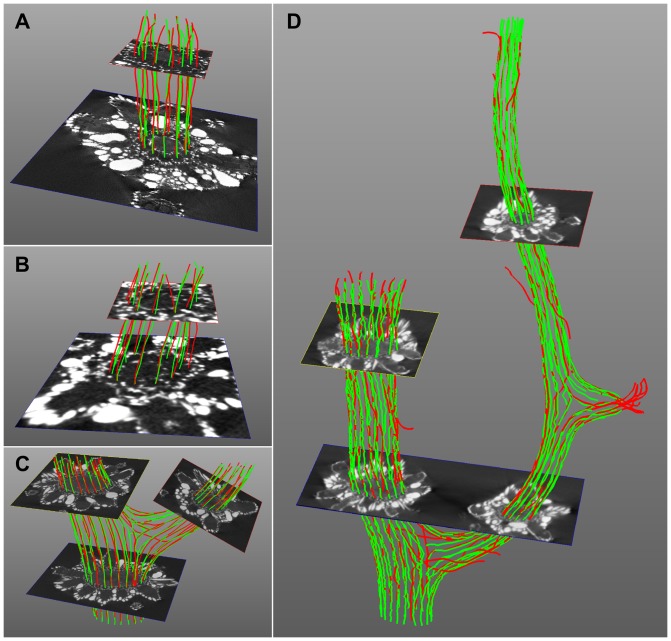
Extracted and reference pathways. The extracted and reference pathways are respectively displayed in red and green, together with the initialization planes in the corresponding datasets: (A) EX, (B) M1, (C) M5, (D) S3.

A total of 132 seed-points located in different branches of the corals were used to extract the canal pathways. As several initialization planes were used in the datasets M5 and S3, the final number of extracted pathways was reduced to 99 after the fusion of curves following the same canal. The details are provided in Table 2. Figure 11 displays the remaining pathways in the datasets evaluated. Most of the extracted pathways (red) closely follow the reference ones (green). This visual impression is confirmed by the quantitative results. Among the 99 extracted pathways 85 were correct (paired with reference pathways) and 14 were erratic, so that the *precision* was 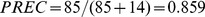, while 10 reference pathways were not paired with any of the extracted pathways, which results in a *recall* equal to 
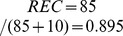
. The details per dataset are displayed in Table 3. These details demonstrate that the average overlap between the paired pathways, measured by the Dice similarity index, ranged between 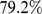 (S3) and 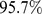 (M5) of the pathway length, with an average of 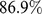. As for the mean distance between the paired pathways, it ranged from 34 to 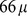
m. The ratio between this distance and the mean estimated canal radius in the given dataset (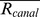) was equal to 0.31 on average.

**Table 2:** Number of seed-points and extracted pathways per dataset.

Image	Seed-points	Pathways after fusion
EX	12	12
M5	50	33
M1	11	11
S3	59	43
**Total**	**132**	**99**

**Table 3:** Results of the pathway extraction.

Image	CORR_E_	ERR_E_	MISS_E_	Dice		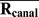 ( 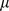 m)	distance ( 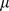 m)	
				mean	std		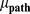	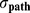
EX	12	0	0	0.869	0.050	201	66	24
M5	31	2	1	0.957	0.051	166	34	12
M1	11	0	0	0.841	0.053	159	60	29
S3	31	12	9	0.792	0.197	116	47	7

### Seed-point localization accuracy

**Figure 12:**
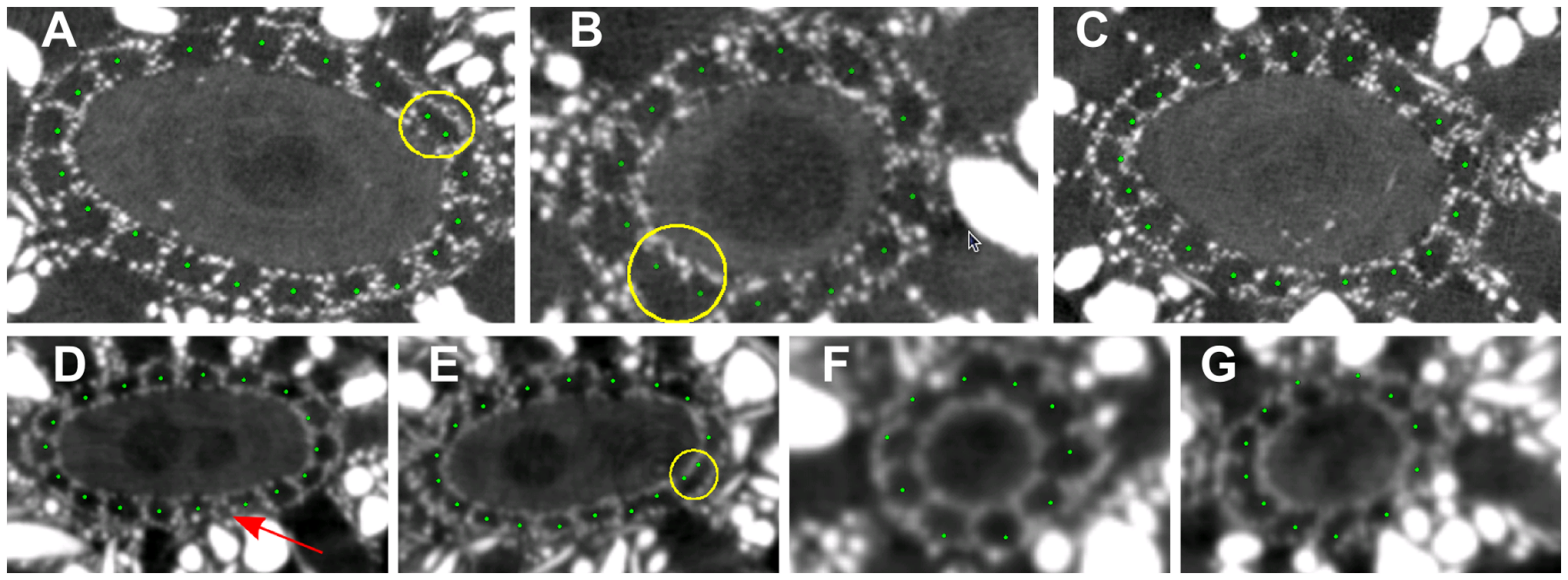
Automatic seed-point initialization. Results of automatic seed-point initialization in M5 (A–C) and S3 (D–G) datasets. Yellow circles highlight the canals where two seeds instead of one were placed. The arrow indicates the missed canal.

**Figure 13:**
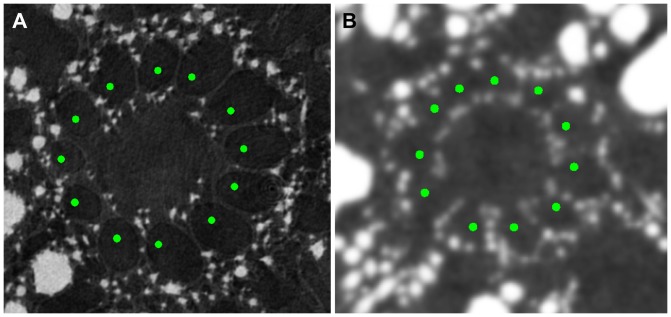
Semi-automatic seed-point initialization in datasets EX (A) and M1 (B).

The results of the seed-point localization algorithm are displayed in Table 4. The seed-points were assigned to a large proportion (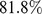) of canals in a fully automatic way and all but one remaining seeds were correctly localized after an elementary user interaction. More precisely, there were 132 canals localized by the observer in the cross-sectional planes considered. The localization was fully automatic (Fig. 12) in 7 out of 9 initialization planes. Among 111 seed-points automatically localized in these 7 planes, 108 were correct and 3 were erroneously placed in already “occupied” canals (Figs. 12(a), 12(b) and 12(e)), although in Figure 12(b) it is actually hard to tell if there is one canal significantly larger than the other ones or two smaller poorly separated canals. One small canal was missed (Fig. 12(d)). In the remaining 2 planes, in datasets EX (Fig. 2(c)) and M1 (Fig. 4(c)), the algorithm failed to delineate the canal region, so a quick user interaction was required to grossly draw the inner boundary of this region and provide its approximate thickness by clicking a point within the outer boundary. After this manual aid, the algorithm automatically found the seed-points in all canals (Fig. 13).

**Table 4:** Results of the automatic seed-point detection.

Image	Plane 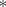	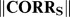	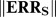	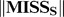	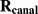 ( 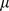 m)	distance ( 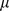 m)	
						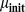	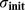
EX	1	12	0	0	201	56	26
M5	1	19	1	0	133	49	22
M5	2	12	1	0	185	49	42
M5	3	19	0	0	180	42	20
M1	1	11	0	0	159	37	13
S3	1	18	0	1	132	45	24
S3	2	18	1	0	127	61	31
S3	3	10	0	0	116	61	24
S3	4	12	0	0	88	51	21

* The location of the initialization planes corresponds to the superimposed lines in Figures 2–5.

**Figure 14:**
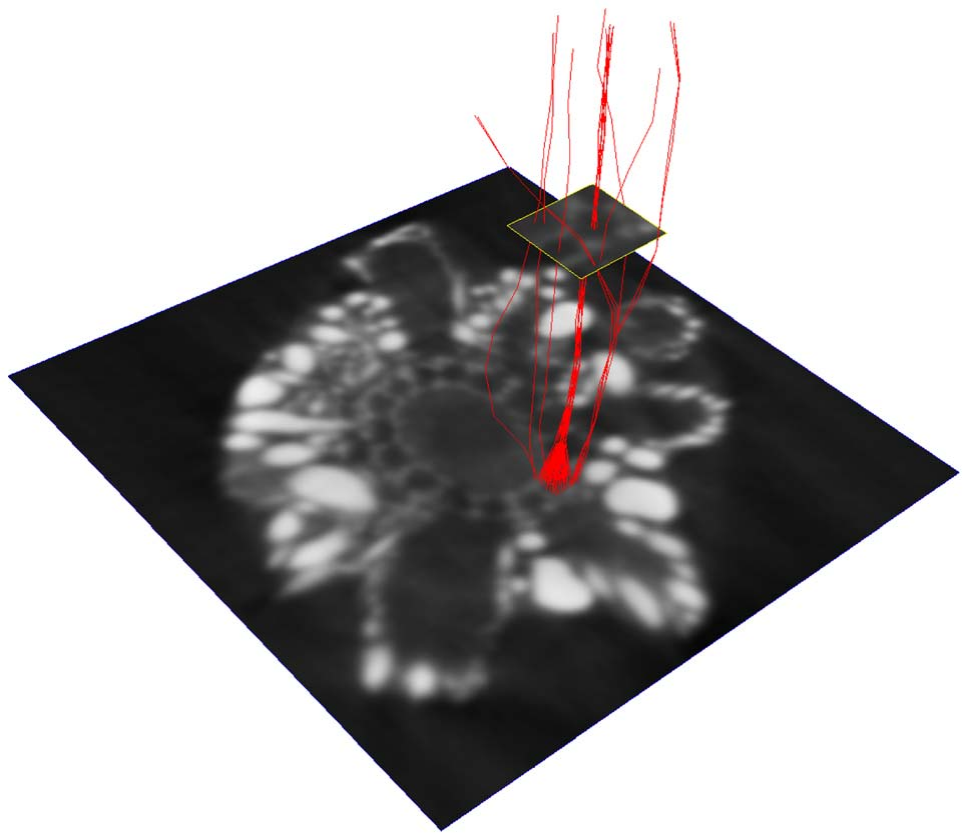
Example of pathways converging from various initialization points. Only a small subset of pathways initialized beyond the canal lumen did not converge.

Considering only the fully automatic localization, the algorithm achieved the following values of precision and recall: 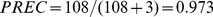, 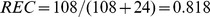, as 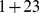 seeds were missing at that stage. Including the semi-automatic localization in EX and M1 datasets, these results respectively increased as follows: 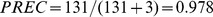, 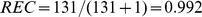. The mean in-plane distance between the calculated seeds and the reference points ranged from 37 to 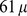
m. The average ratio between this distance and the corresponding estimated canal radius (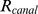) was equal to 0.36. It can therefore be concluded that the automated localization of seed-points was accurate, as the seeds were located close to the canal center: at a distance, on average, smaller than half the canal radius. As for the small number of seed-points generated farther from the canal centers, our experience shows that a poorly centered initialization generally does not prevent the pathway from converging towards the canal lumen (Fig. 14).

## Discussion

The goal of our study was and remains to understand the internal organization of the octocoral stem canals, as well as their physiological and functional role in the growth of the colonies. A crucial step towards this goal was to prepare appropriate non-destructive methods giving access to the internal ultrastructure and organization of the network. We focused our effort on imaging tools, namely 3D image acquisition and image processing, with a particular emphasis on automated extraction of canal pathways.

### On image acquisition

After several attempts, we converged towards an acquisition protocol giving satisfactory results (see settings in Table 1), although the number of tested configurations was not large enough to claim that these settings actually were optimal. To the best of our knowledge, thus acquired images are the first 3D representation of the octocoral internal structure. Despite the small number of exploitable datasets acquired to date, our images were quite diversified: four different spatial resolutions ranging from 4.5 to 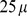
m, topology ranging from simple segments to several branches with bifurcations, apical segments, a “bud” of a new branch. Additionally, these datasets contained more than hundred different canals. The range of spatial resolutions explored was selected considering the size of the structures of interest. As expected, the finest resolution (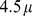
m) gave the most detailed insight to the internal structure. However, to obtain a good quality image at such a resolution, the acquisition has to be performed very carefully, with a perfect immobilization of the sample. Furthermore, with a constant size of the imaging sensor, the image resolution is proportional to the field of view. So, at very fine resolution, FOV is strongly reduced. Consequently, the number of acquisitions necessary to cover a whole sample should rise considerably, thus increasing the total time and cost. From our first experience, spatial resolutions between 10 and 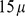
m currently represent the best trade-off. Concerning the conditioning of the samples, the immobilization is the condition number one of successful acquisitions. The solution adopted, which uses tubes, is only partly satisfactory, as it leads to cutting the salient branches. Further investigation is needed to find solutions less invasive for specimens.

We have chosen to use a micro-CT imaging device, as it offers a very fine spatial resolution (a few microns) and acceptable acquisition time (below one hour). Additionally, from medical imaging experience, CT is well suited to visualize calcified structures [34]. The drawback of this imaging modality is its difficulty to differentiate soft tissues, particularly in the presence of the so-called blooming effect that is also observed in medical imaging in patients with strongly calcified arteries [35]. This effect is due to beam alteration when traversing high-density calcified structures. At the initial stage of this study we also acquired various 3D images using a high-resolution magnetic resonance imaging (MRI) system in an attempt to depict soft tissues in the octocorals. Contrary to CT imaging, MRI is not suitable to visualize calcareous structures that appear black in these images. Furthermore, the acquisitions were much more time-consuming and the spatial resolutions obtained were between two and three times grosser than in micro-CT. Both modalities are costly and neither can acquire the images *in situ*. The modality best suited to acquire images in a water tank is ultrasound. This modality is also the cheapest one and high-frequency transceivers might be able to achieve an appropriate spatial resolution. However, in this modality calcified structures are very likely to hamper the visualization of the interior of the corals. Moreover, obtaining a 3D representation of the samples would be much less straightforward than with CT and MRI. For similar reasons, optical imaging would be unsuited to study *Muricea Muricata*, although it was successfully used in [36] to quantify anthozoan gastrovascular flow *in vivo* in a stoloniferan octocoral referred to as clavulariid species A, which is characterized by a complete absence of sclerites.

### On image processing

**Figure 15:**
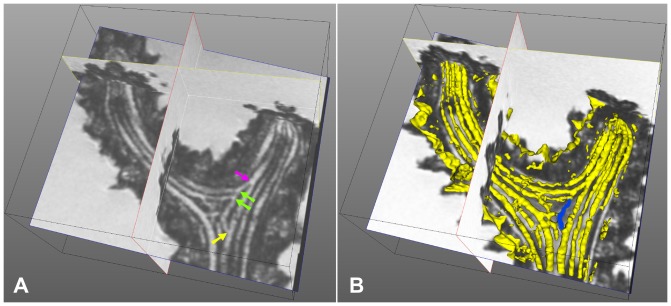
Example of a complex canal configuration in a branching region. (A) longitudinal slightly oblique slice from the original image, (B) attempt to 3D surface rendering of the canals present in this region. A canal bifurcates (yellow arrow) giving rise to a secondary pathway that successively joins two canals coming from a colony branch (green arrows) and finally joins the principal canal again (magenta arrow). The secondary pathway highlighted in blue on the 3D rendering cannot be extracted with the current version of our method, but was noticed when visually following the extracted pathways.

When dealing with 3D images, the external structure of objects can be assessed using relatively standard software tools such as thresholding, mathematical morphology and surface rendering [17], [18]. It is much more difficult to assess the internal structure, and the development of dedicated image-processing algorithms is needed prior to the application of more standard visualization and measuring tools. For the purpose of our study, we developed such specific methods. The main contribution was an algorithm capable of automatically tracking the pathway of a selected canal. Another useful algorithm automatically places seed-points in each canal perceptible in a selected cross-sectional plane, in order to initialize the pathway tracking. Our algorithms consistently achieved detection rates and overlap values greater than 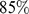, with distances to the reference of the order of one third of the estimated canal radius, on average. These results seem quite good, but cannot be compared to the literature, as no sufficiently similar work has been published yet. Therefore, our work sets a benchmark in this field. An interesting extension might be an automatic detection of canal bifurcations to locally reinitialize the tracking process and extract the canal sections that do not exist in the whole extent of a branch (Fig. 15).

The measure of overlap between the extracted pathways and the reference ones depends on the criterion defining the true positive, false positive and false negative points. With a criterion based on a maximum distance 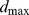, equal to the estimated average radius of canals, the pathway points located at less than 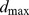 from the reference pathway are expected to fall within the canal “lumen”. However, one can argue that a criterion only based on distance is somewhat arbitrary, as the dimensions of canals may vary along the pathways. In particular, they may increase significantly near bifurcations, where an extracted pathway may be located far from the reference pathway, while remaining correct. Unfortunately, the criterion used in (File S1, Section 2.2) to automatically check whether or not two points are located within the same canal could not be used in this case. Indeed, due to apparent “holes” observed in the canal walls, some points might be falsely considered as located in the same canal. This was not a problem when searching for twin pathways, as the criterion was applied globally. Here though, it would artificially enhance the true positive score. Moreover, visually assessing, for each point of each extracted pathway, whether or not it falls in the same canal as the corresponding reference pathway, would be excessively tedious. Therefore a distance criterion equal to the estimated canal radius seems to be a reasonable trade-off.

The parameter settings were empirically fixed during the development, based on limited training data. These settings may potentially be suboptimal and their sensitivity was not rigorously assessed. Nevertheless, we believe that the algorithms are relatively robust to the parameter settings, since they achieved consistent scores in images having a wide range of characteristics (resolution, contrast, anatomical complexity), as stated in the previous section.

### On structural observations

Our previous understanding of the stem canal network in octocorals was rather simplistic, in short: the number of canals decreases linearly towards the branch tips [7]. Although this pattern holds if one examines the number of canals every certain distance, in this study we clearly noticed that new canals can arise at several parts of the network. Actually, several canal patterns were observed. Although many canals were simple, *i.e.* only followed the main branch or “turned” into a secondary branch, many others bifurcated or fused. Particularly noteworthy, branching points consistently showed canal bifurcations allowing the original canal to continue towards the tip but maintaining a connection with the new branch. This bifurcation process was also consistent with an increase in the canal dimension as a preamble to division. These observations answer our former question on the relationship of the canal network and branching. This also opens the possibility that canal bifurcation at branching points can be modeled with respect to the ratio between mother and daughter branches likewise many other aspects of gorgonian form and growth [5]. These observations also contribute to the notion of rapid connectivity within gorgonian coral colonies as experimentally observed using labeled elements in *Pseudoplexaura porosa* [37]. It is unknown, however, the reason why some canals appeared (mainly but not only by bifurcation) or ended (mainly by fusion) anywhere along a branch.

After examining the structure of the gorgonian stem canal network with high-resolution X-ray computed tomography imaging the growth of the network, as well as of the colony, appears as a more complex process than previously thought. It was unknown if sites in the colony producing new canals could be either at the colony base, at the branch tips or both. Now it is clear that a significant number of canals originate at branching points, which is a process that should reiterate when the first polyp started to multiply and form the primary branch. Here we observed that stem canals fused at the branch tips, forming a unique chamber of complex architecture. In the analyzed apical regions of *Muricea muricata* the number of canals just before fusion varied from 10 to 12, whereas in another gorgonian species, *Eunicea mammosa*, the minimum number of canals found in a number of dissected colonies was sixteen, a multiple of eight [7]. Based on that previous observation, it was expected that the number of canals at the tips is connected and related to the number of polyp mesenteries, which is always eight in octocorals [8]. Branching in octocorals has different levels of complexity from very simple, nearly unbranched colonies, to repeatedly branched candelabrum- and mesh-like seafans. Early diverged octocorals with very simple colonies, such as members of family *Telestidae* [38], could bring some evidence to this discussion. Telestids (*e.g.*, *Telesto* and *Carijoa*) form hollow tubes emerging from stolons, which have an apical polyp with subordinate lateral polyps [39]. All telestid polyps are connected to the same fluid in the tube. The walls of the colony have the same eight grooves from the primordial apical polyp. Moreover, this suggests that despite the simplicity of these colonies, the main branch retains features of the eight mesenteries of the first polyp. Likewise, gorgonian colonies might start growing formed by the extension of mesenterical structures.

This study answered some questions on the nature and organization of the pseudovascular tree-like network of a colonial animal, but raised new ones. The imaging method tested here opens a number of applications and possibilities to study important biological and environmental questions for coral biology. The non-destructive approach is likely to make the method suitable for diverse *in vivo* assays. For instance, mesocosm ocean acidification and calcification experiments, which are in great need of methods providing fine resolution images of growth and calcification [40], might make use of this approach. In theory, the specimens could be moved to a nearby micro-CT device for image acquisition and then back to the experimental container, without damage to the experimental corals. Mesocosm ocean acidification experiments are meant to test the effects of high pCO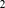 on marine calcifying organisms (*e.g.*, [41]). Currently, this kind of experiments require destructive analysis to measure the fluorescent incorporation of labeled calcium and extended periods of confocal microscopy to estimate calcium incorporation at many sclerites. We were surprised to see the fine detail and resolution of the stem canal wall of *Muricea muricata*, which is made of calcite sclerites. With a micro-CT approach similar to the one developed here, one might examine a large extent of canals and other calcifying structures as a measure of dissolution, for instance, following the same specimen multiple times along the experiment. This would open an entirely new era of non-destructive possibilities for growth and calcification experimentation in corals.

In summary, our work not only contributes to better understand the internal organization of octocoral tree-like networks but provides a non-destructive computerized method to examine the skeletal ultrastructure of octocorals, which has many applications. Using the methods developed here we can reconstruct the pathway of each stem canal, with minimum user interaction. Given the small size (micrometers) of the structures to analyze, high resolution X-ray computed tomography images were needed. To the best of our knowledge the images acquired within this study belong to the first attempts to assess the internal structure of canals in octocorals by means of micro-CT, which demonstrated to be feasible without the need of histological or other destructive methods. Using our method applied onto these images, a 3D representation of the canal network was generated for the first time. Results that otherwise would have needed a great deal of serial histological sectioning, which damages the specimens and involves fixation artifacts.

## Supporting Information

Figure S1
**Delineation of the canal region.** (A) Cross-sectional image. (B) Region 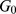 superimposed onto the cross-section, as well as the extracted external and internal contour of the canal region. (C) Ring enclosing the canal region. (D) Variance 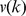 of the gray levels on the “deflating” contours as a function of 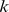, which denotes the number of iterations (erosions).(TIFF)

Figure S2
**Quasi-periodic pattern of the canal region.** (A) Unrolled canal region 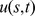 from the cross-section corresponding to Fig. 1(c). (B) Signal 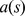 calculated according to (Eq. 1, File S1) in this region.(TIFF)

Figure S3 **Low-pass filtering in the frequency domain.** (A) Fourier transform of the original signal. (B) Original and filtered signal.
(TIFF)

Figure S4
**Detecting points located in different canals.** (A) Cross-sectional image with a pair of points located in different canals. (B) Image intensity profile between these points. The dashed line corresponds to the threshold equal to 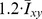, where 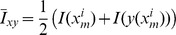.(TIFF)

Figure S5
**Definition of true positive, false positive and false negative points.** True positive (TP), false positive (FP) and false negative (FN) points are labeled based on distances between the corresponding points in the extracted pathway 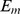 and reference pathway 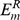. True positives are points in 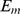 located at distances smaller than the threshold 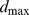 from 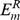, whereas false positives and false negatives are points located in respective sections of 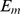 and 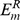, where the distance between these pathways is larger than 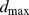.(TIFF)

File S1 **Appendix.** Automated determination of the seed points. Post-processing. Pathway evaluation measures.
(PDF)
