# Supplementary figures and images for: A Semi-Automatic Method to Extract Canal Pathways in 3D Micro-CT Images of Octocorals
Source: PLoS One. 2014 Jan 23;9(1):e85557. doi: 10.1371/journal.pone.0085557 (PMC3900427; doi:10.1371/journal.pone.0085557)

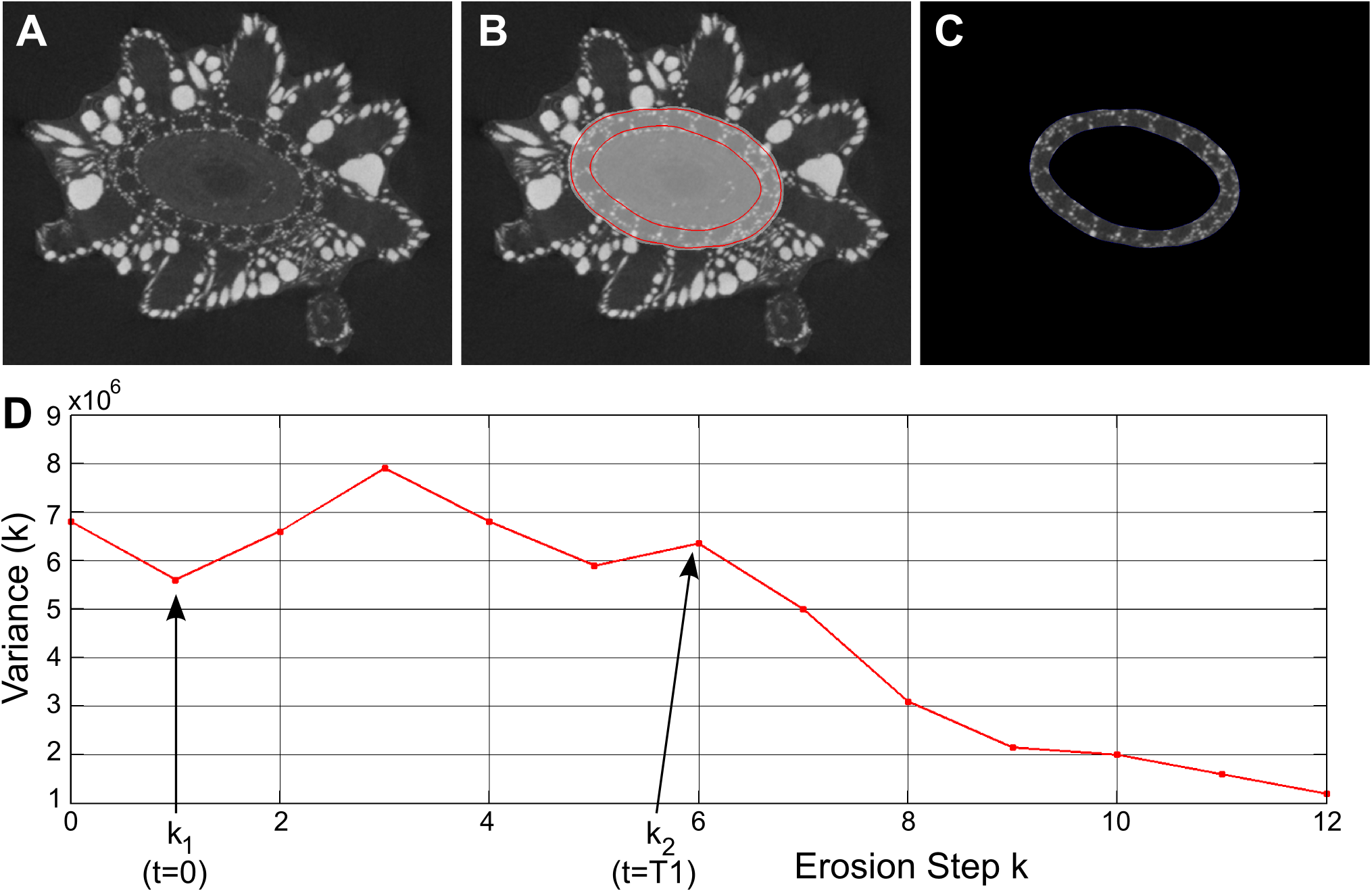

Supplement: Figure S1 — Delineation of the canal region. (A) Cross-sectional image. (B) Region superimposed onto the cross-section, as well as the extracted external and internal contour of the canal region. (C) Ring enclosing the canal region. (D) Variance of the gray levels on the “deflating” contours as a function of , which denotes the number of iterations (erosions). (TIFF) [file pone.0085557.s001.tiff]

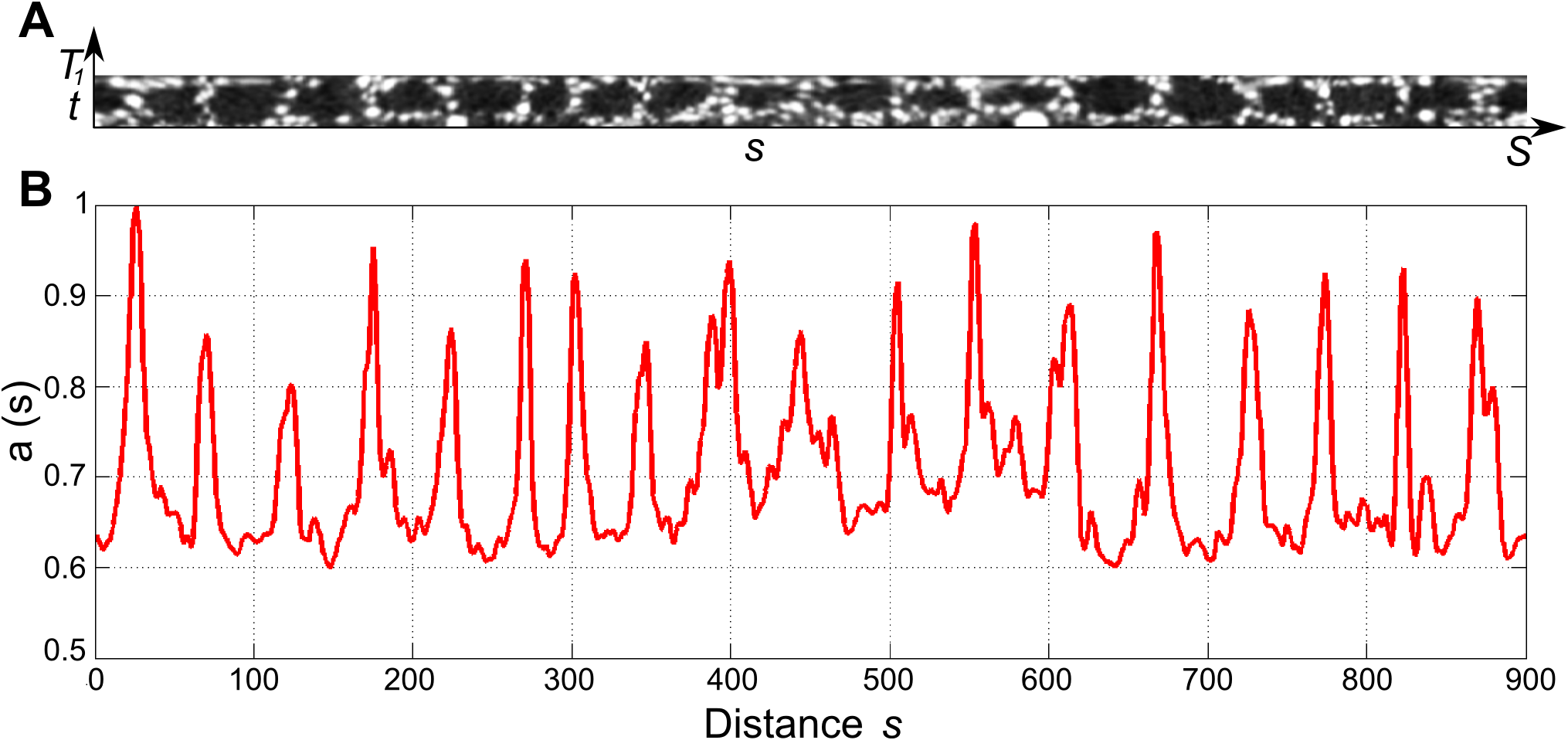

Supplement: Figure S2 — Quasi-periodic pattern of the canal region. (A) Unrolled canal region from the cross-section corresponding to Fig. 1(c). (B) Signal calculated according to (Eq. 1, File S1) in this region. (TIFF) [file pone.0085557.s002.tiff]

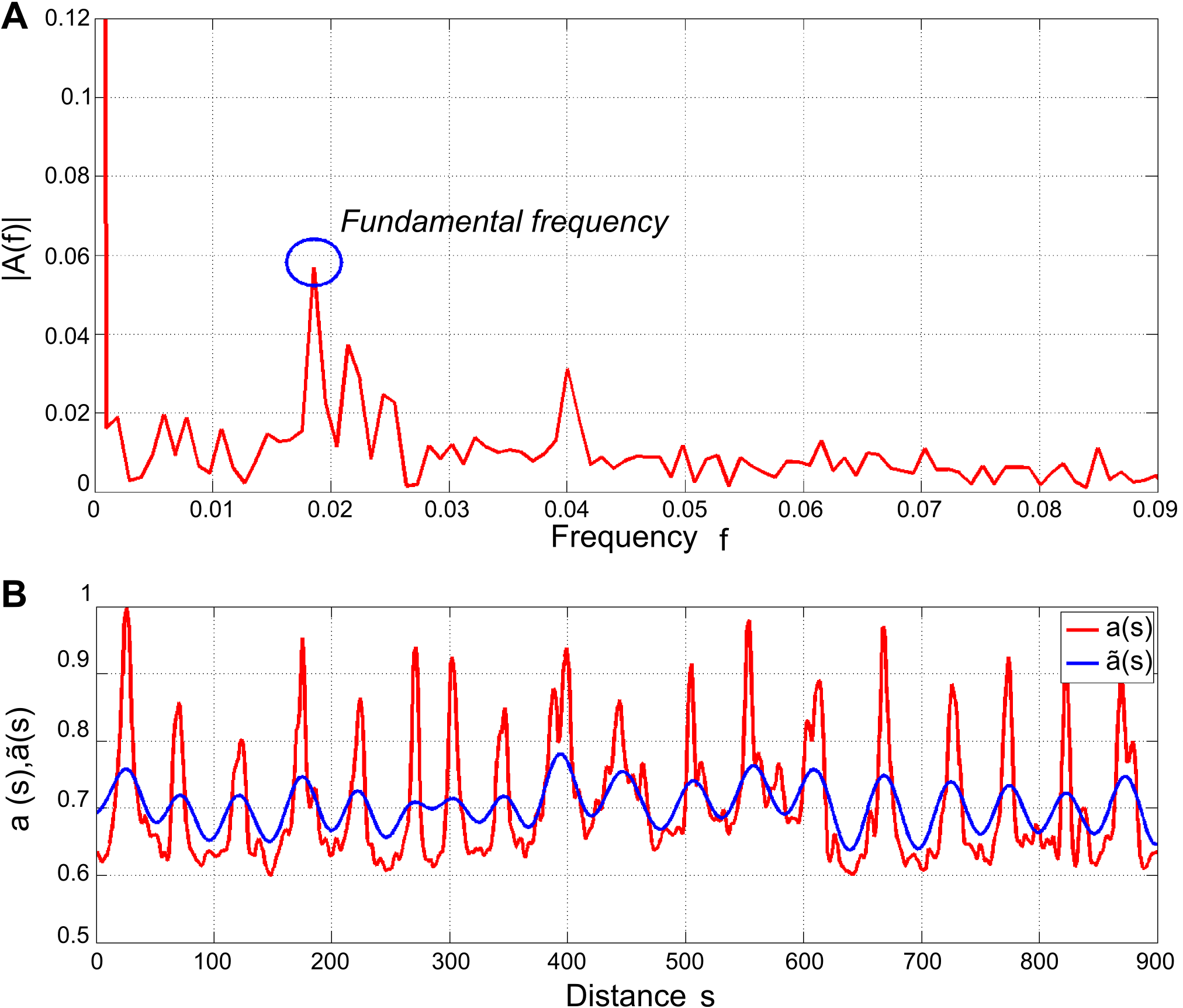

Supplement: Figure S3 — Low-pass filtering in the frequency domain. (A) Fourier transform of the original signal. (B) Original and filtered signal. (TIFF) [file pone.0085557.s003.tiff]

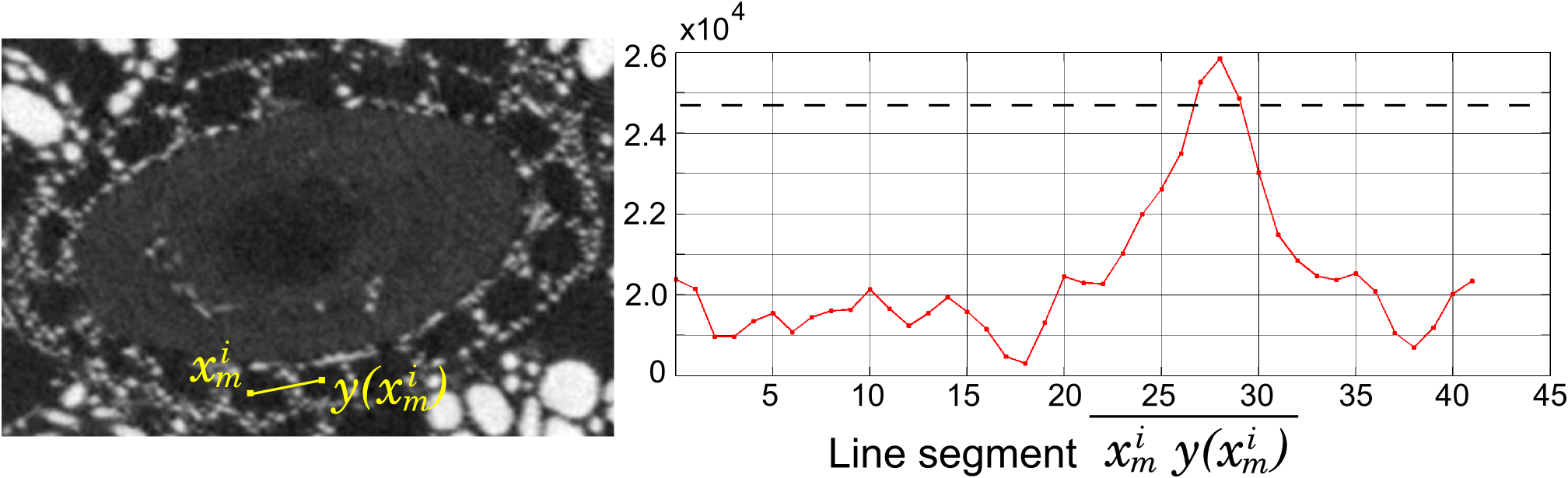

Supplement: Figure S4 — Detecting points located in different canals. (A) Cross-sectional image with a pair of points located in different canals. (B) Image intensity profile between these points. The dashed line corresponds to the threshold equal to , where . (TIFF) [file pone.0085557.s004.tiff]

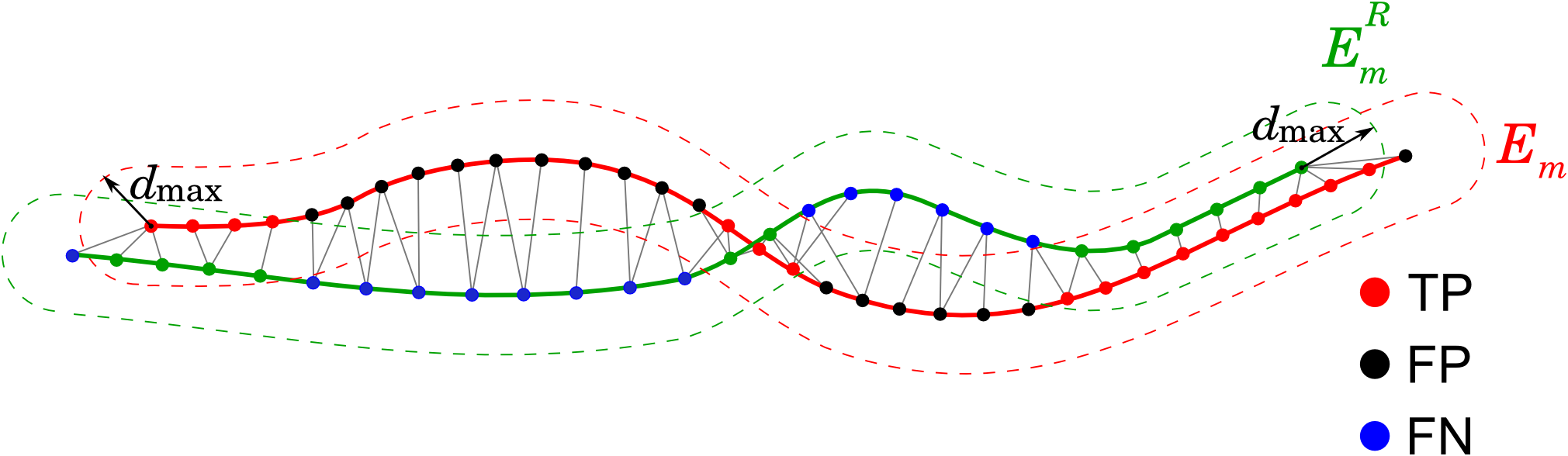

Supplement: Figure S5 — Definition of true positive, false positive and false negative points. True positive (TP), false positive (FP) and false negative (FN) points are labeled based on distances between the corresponding points in the extracted pathway and reference pathway . True positives are points in located at distances smaller than the threshold from , whereas false positives and false negatives are points located in respective sections of and , where the distance between these pathways is larger than . (TIFF) [file pone.0085557.s005.tiff]
