# Supplementary material for: A Semi-Automatic Method to Extract Canal Pathways in 3D Micro-CT Images of Octocorals
Source: PLoS One. 2014 Jan 23;9(1):e85557. doi: 10.1371/journal.pone.0085557 (PMC3900427; doi:10.1371/journal.pone.0085557)
Supplement: File S1 — Appendix. Automated determination of the seed points. Post-processing. Pathway evaluation measures. (PDF) [file pone.0085557.s006.pdf]

Appendix

1 Automated determination of the seed points

After extracting a cross-sectional image locally perpendicular to a branch, the algorithmic steps are:

1. rough delineation of the gorgonin axis;
2. extraction of the internal and external boundary of the region (ring) containing the canals;
3. localization of seed points within the canal region.

1.1 *Approximate delineation of the gorgonin axis region*

Let I^j denote the current (j-th) cross-sectional image (Fig. S1.A) and O^j denote a seed point located within the gorgonin axis in I^j. First, the image-gradient magnitude in I^j is calculated and thresholded with a predefined threshold. Binary edges thus obtained subsequently undergo a morphological closing operation in order to fill the gaps. Then, the region G^j composed of all the connected pixels located between the edges and the point O^j is segmented out. In case of an unsatisfactory result, the user can interactively adjust the threshold thanks to a dynamic display and a user-friendly graphical interface.

1.2 *Delineation of the canal region*

The segmentation of the gorgonin axis region in the previous step may lead to leakages into some canals, owing to "holes", *i.e.* local lack of contrast between these canals and the gorgonin axis. Here we attempt to delineate the 2D boundary between the gorgonin axis and the canals more accurately, as well as to localize the external boundary of the canal region. The region G is first strongly dilated (from here forth, we drop the superscript j in order to simplify the notations), so as to make its boundary smoother and to "push" its boundary beyond the canal region. To this purpose, the size of the structuring element (disc) is set slightly greater than the typical diameter of the canals and depending on the image resolution. Let G_0 and B_0 respectively denote thus dilated region and its boundary (Fig. S1.B). The actual boundaries of the canal region are identified by iteratively "deflating" the contour B and analyzing the image intensities of the pixels that compose B. The "deflation" is actually obtained by eroding the region G, starting from G_0, with a structuring element defined as a disc with a radius equal to one pixel. The iterations are numbered using a subscript k, so that G_k is the region resulting from the k-th erosion and B_k is its boundary. We define $v(k)$ as the variance of the gray level values of the pixels forming B_k. As long as the boundary B_k is beyond the canal region, the variance $v(k)$ is large due to the presence of heterogeneous structures such as sclerites, polyps, etc. Conversely, when the boundary B_k falls within the gorgonin axis, the variance $v(k)$ is small. In-between, *i.e.*, when the boundary falls within the canal region, the variance $v(k)$ takes intermediate values varying irregularly. Our algorithm first approximately identifies the external boundary of the canal region as the one corresponding to the first minimum ($k = k_1$) in the curve $v(k)$ and the internal boundary as the last maximum ($k = k_2$) in this curve (Fig. S1.D). Then, to make the external contour smoother, the corresponding region G_{k_1} is blurred with a Gaussian filter and a new boundary $\overline{B_{k_1}}$ is extracted by means of an isocontour defined by an isovalue equal to 0.5 (Fig. S1.B). The final internal contour $\overline{B_{k_2}}$ is placed at a constant distance $T_1 = k_2 - k_1$ inwards from $\overline{B_{k_1}}$. In case of failure the user can either interactively choose a different cross-section and run the process again, or manually draw the contours.

1.3 *Seed-point localization in each canal*

By construction, the canal region extracted forms a ring with constant thickness T_1. If the ring is cut at any point orthogonally to the contours, and the curvilinear coordinate on the external contour is used as abscissa s, then the canal region can be transformed into a rectangle (Fig. S2.A) by an appropriate resampling along inwards-directed lines orthogonal to the external contour. This rectangle will be referred to as *unrolled* image $u(s,t)$, where the ordinate t corresponds to the distance from the external contour, *i.e.*, $t = 0$ corresponds to $k = k_1$ and $t = T_1$ to $k = k_2$ (Fig. S1.D). The canal region displays nearly periodic structures that define the canals and their walls. This periodicity is captured by calculating a signal a (Fig. S2.B) such that each value $a(s)$ is a weighted mean gray-value of the column s in $u(s,t)$, *i.e.*:

$$a(s) = \frac{1}{T_1} \sum_{t=0}^{T_1-1} \beta(t) u(s,t), \forall s \in \{0, \ldots, S-1\}, \tag{1}$$

$$\text{where}\, \beta(t) = 1 - \frac{|t - T_1/2|}{T_1/2},$$

and S is the number of points in the external contour. The weighting coefficients $\beta(t)$ were defined in such a way that the highest weights are assigned to the points located far from the boundaries of the canal region. At these locations the largest and most regular gray-value variations from one canal to another are observed. The deep valleys in the graph representing the signal $a(s)$ correspond to the canals. So the idea is to place the seed points in these valleys. However, the signal $a(s)$ is noisy and we propose to reduce the noise level by smoothing it with an adaptive low-pass filter. The filtering is performed in the Fourier domain. The Fourier transform $\mathcal{A}$ of the signal a is calculated in order to identify the fundamental spatial frequency $f = f_0$ (Fig. S3.A), which characterizes the periodicity of the pattern of interest. This frequency corresponds to the most prominent peak in $\mathcal{A}(f)$, discarding the one at $f = 0$. After the identification of the peak at f_0, the following low-pass filter is used:

$$H(f) = \begin{cases} \mathcal{A}(f), & \text{if } f \leq f_0 \\ \mathcal{A}(f)(-2\frac{f}{f_0} + 3), & \text{if } f_0 < f \leq 1.5 f_0 \\ 0, & \text{if } f > 1.5 f_0. \end{cases} \tag{2}$$

Then the inverse Fourier transform is used to reconstruct the smoothed signal $\tilde{a}$ (Fig. S3.B). The abscissa values s corresponding to the valleys in $\tilde{a}(s)$ are stored and used to calculate the seed locations. The ordinate used to calculate the seeds is $t_0 = T_1/2$. Thus calculated initial points fall within the canals and are used to fix one end of the cylinder axis of C_{in} and C_{out}.

2 Post-processing

2.1 *Detecting and cutting incorrect pathways*

The first step is to find, for each pathway $E_m \in \mathcal{E}, m \in \{1, 2, ..., M\}$, its nearest neighbor $E_{m'} \in \mathcal{E}$, where $\mathcal{E}$ denotes the complete set of M pathways extracted from a given image ($M = |\mathcal{E}|$). Let $E_c \in \mathcal{E}$ denote a candidate pathway, with $c \in \{1, 2, ..., M\} \setminus m$. For each point $x_m^i \in E_m$, the distance $\delta(x_m^i, E_c)$ to the nearest point in the candidate pathway E_c is calculated. We denote this nearest point $y_c(x_m^i) = \operatorname*{argmin}_{x \in E_c} \left(\delta(x_m^i, E_c)\right)$, whereas $\hat{\delta}_{mc} = \max_{i=1,2,\ldots,|E_m|} (\delta(x_m^i, E_c))$ denotes the largest local distance between E_c and E_m, *i.e.*, the largest distance between paired points $x_m^i \in E_m$ and $y_c(x_m^i) \in E_c$ across all possible values of i. $E_{m'}$ corresponds to the candidate pathway such that $\hat{\delta}_{mc}$ is minimum: $E_{m'} = \operatorname*{argmin}_{E_c} \left(\hat{\delta}_{mc}\right)$.

Once the nearest pathway $E_{m'}$ assigned, the average point-wise distance between the pathways E_m and $E_{m'}$ is calculated: $\bar{\delta}_m = \frac{1}{|E_m|}\sum_{i=1}^{|E_m|}\delta(x_m^i, E_{m'})$. After having done it for all $E_m, m = 1, 2, \ldots, M$, the mean value $\mu_\delta = \frac{1}{M}\sum_{i=1}^{M}\bar{\delta}_m$ and the corresponding standard deviation σ_δ are calculated. The pathways to be cut are those that meet the following criterion: $\bar{\delta}_m > \mu_\delta + \sigma_\delta$. However, the statistics μ_δ and σ_δ may be biased by the outliers. Therefore, the pathways to be cut are removed from $\mathcal{E}$, in order to iteratively recalculate μ_δ and σ_δ, as long as no more pathways to be cut are detected.

Cutting thus detected possibly erratic pathways is based on the statistics of the image intensity within the canals. It is expected, that the correct part of the pathway has the intensities close to the average in-canal intensity. We calculate both the mean value μ_{canal}, by averaging all the values μ_{in} (mean gray level within the optimally oriented cylinder C_{in}) obtained along all the canals, and the corresponding standard deviation σ_{canal} of these values. Then the value of μ_{in} in each point of each pathway that needs to be cut is compared to these statistics and the corresponding point is labeled as *correct* if μ_{in} falls within the interval $\mu_{\text{canal}} \pm 2\sigma_{\text{canal}}$ or *incorrect* otherwise. Subsequently, in each of these pathways, the longest segment of consecutive *correct* points is retained, whereas the remainder is cut. Actually, up to three consecutive *incorrect* points are tolerated within the segment that is retained, so as to cope with local variability.

2.2 *Fusion of twin pathways*

At branching points, some canals follow the main branch, others turn towards the secondary branch, whereas the remaining ones bifurcate and follow both branches. Therefore, it is necessary to initialize the pathway extraction in each branch, so as to maximize the number of pathways extracted. However, this leads to multiple pathways that may follow the same canal. As it is preferable to have one pathway per canal, the twins have to be detected and combined. To do so, we check whether or not two nearest pathways are separated by a canal wall.

Let E_m and $E_{m'}$ be the neighboring pathways, and $y(x_m^i) \in E_{m'}$ the nearest point to $x_m^i \in E_m$ determined as described in the previous paragraph. For each pair $x_m^i, y(x_m^i)$, a straight line segment is drawn between these points, and the profile of image intensity I along this segment is analyzed (Fig. S4). We consider that the two points lie in different canals (*i.e.*, are separated by a canal wall), if an intensity increase of at least 20% is observed along the profile with respect to the average gray-level of its endpoints $\bar{I}_{xy} = \frac{1}{2}\left(I(x_m^i) + I(y(x_m^i))\right)$. If more than 90% of pairs fall within the same canal, the corresponding pathways are considered as twins. Their common part is therefore to be averaged, while the differing extremities are kept unchanged.

3 Pathway evaluation measures

The process begins by equidistantly sampling the pathways (distance between samples equal to three times the voxel size) to make the comparison more accurate. The following steps are then executed:

- For each reference pathway E_m^R, assign the extracted pathway visually corresponding to the same canal, and label the pathways as follows: mark as missed ($MISS_E$) the unpaired reference pathways, mark as erratic (ERR_E) the unpaired extracted pathways, and mark as correct ($CORR_E$) the paired pathways.

- For each point x_m^i of each paired pathway E_m, find the nearest point y_m^i in the corresponding reference pathway E_m^R, using the function $\delta(x_m^i, E_m^R)$ defined in 2.1. Symmetrically, for each point in E_m^R, find the nearest point in E_m. Each point of the extracted pathway is thus connected to at least one point of the corresponding reference pathway and *vice versa* (Fig. S5).

- If more than one point in E_m is connected to an endpoint of E_m^R, clip the surplus points and discard them in the evaluation. This step was performed only at some locations, where the manual tracing of the pathways was too difficult: the observer preferred to leave the reference pathways shorter, but extra segments of the extracted pathways could not be labeled as true or false.
- Label the remaining points as follows: mark points in the extracted pathways ($x_m^i \in E_m$) as true positives (TP), if they have at least one corresponding point $y_m^i \in E_m^R$ located at a distance $\delta(x_m^i, E_m^R) < d_{\max}$, as false positives ($FP$) otherwise; mark as false negatives (FN) the points in reference pathways ($y_m^i \in E_m^R$) such that the corresponding extracted pathway is too far: $\delta(y_m^i, E_m) \geq d_{\max}$.
- Calculate the mean μ_{path} and standard deviation σ_{path} of the distances $\delta(x_m^i, E_m^R)$ for the points x_m^i labeled as true positives.

The maximum distance $d_{\max}$ was set equal to the estimated canal radius averaged in the given dataset, *i.e.*: $d_{\max} = \overline{R_{canal}}$. With thus assigned labels, we defined the measures that summarize the proportions between the cardinalities of the label sets, namely precision, recall, and Dice similarity index:

$$DICE = \frac{2 \cdot ||TP||}{2 \cdot ||TP|| + ||FN|| + ||FP||}. \tag{3}$$
